# Linking artificial sweetener intake with kidney function: insights from NHANES 2003–2006 and findings from Mendelian randomization research

**DOI:** 10.3389/fnut.2024.1387676

**Published:** 2024-05-30

**Authors:** Zhuoling Ran, Yuxuan Zheng, Lin Yu, Yuxian Zhang, Zhenjiang Zhang, Huijie Li, Xuhan Li, Jing Song, Li Zhang, Ran Zhang, Chang Lu, Yang Gong, Jian Gong

**Affiliations:** ^1^Research Group of Jian Gong on Pharmacoepidemiology and Clinical Drug Evaluation, School of Life Science and Biopharmaceutics, Shenyang Pharmaceutical University, Shenyang, China; ^2^McWilliams School of Biomedical Informatics, University of Texas Health Science Center, Houston, TX, United States

**Keywords:** artificial sweeteners, kidney function, chronic kidney disease, National Health and Nutrition Examination Survey, Mendelian randomization

## Abstract

**Background:**

The current investigation examines the association between artificial sweetener (AS) consumption and the likelihood of developing chronic kidney disease (CKD), along with its impact on kidney function.

**Methods:**

We utilized data from the National Health and Nutrition Examination Survey from 2003–2006 to conduct covariance analysis and weighted adjusted logistic regression, aiming to assess the association between artificial sweetener intake and CKD risk, as well as kidney function indicators. Subsequently, we employed Mendelian randomization methods to validate the causal relationship between the intake of artificial sweeteners, CKD risk, and kidney function indicators. Instrumental variable analysis using inverse-variance weighting and Robust adjusted profile score were the primary analytical methods employed.

**Results:**

A total of 20,470 participants were included in the study, with 1,257 participants ultimately included in the analysis. In all adjusted logistic regression models, no significant association was found between the intake of artificial sweeteners and CKD risk. Similarly, the summary odds ratios (OR) for each unit change in genetically predicted CKD risk were 2.14 (95% CI: 0.83, 5.21, *p* = 0.092), 1.41 (95% CI: 0.54, 3.63, *p* = 0.482), and 1.50 (95% CI: 0.50, 4.52, *p* = 0.468) for the impact of artificial sweeteners added to cereals, tea, and coffee, respectively. It was only observed that adding artificial sweeteners to coffee was associated with a modest reduction in urinary albumin-to-creatinine ratio (OR = 0.94, 95% CI: −0.108, −0.022, *p* = 0.003), the effect appeared to be relatively small and may not directly impact the individual level.

**Conclusion:**

Our study does not support a causal relationship between artificial sweetener intake and the risk of CKD. However, due to the limitations and potential confounding factors, these findings need to be further validated through larger sample sizes in observational studies and Mendelian randomization analyses.

## 1 Introduction

Chronic kidney disease (CKD) has emerged as a substantial global public health issue, marked by its elevated prevalence, disability rates, substantial healthcare expenses, and limited awareness often referred to as the “triple high and one low” (1). According to the International Society of Nephrology’s Global Kidney Health Atlas, the global median prevalence of CKD in 2023 is 9.5%, and the median proportion of deaths due to chronic kidney disease is 2.4% (2). Projections suggest that by 2040, CKD will rank as the fifth leading cause of death worldwide (3, 4). CKD is characterized by a severe decline in kidney function, reduced glomerular filtration rate (GFR), heightened urinary albumin excretion (proteinuria), and confirmed by two or more diagnoses at least 3 months apart (5). The condition is closely associated with various comorbidities, including diabetes (6, 7), hypertension (8, 9), obesity (10, 11), particularly cardiovascular diseases (12), and is strongly correlated with an increased all-cause mortality rate (13, 14). Beyond significantly impacting patients’ quality of life, CKD also stands as a major contributor to poverty. With a growing focus on health and overall well-being in recent decades, individuals have become increasingly conscious of minimizing their intake of high-sugar, high-salt, or high-fat foods. Alongside the growing interest among consumers in reducing sugar intake, the consumption of foods containing zero-calorie substitutes (artificial sweeteners) has become increasingly popular (15). They are often used as a supposedly healthier or “diet-friendly” alternative. With the sharp increase in the consumption of products containing artificial sweeteners, concerns have arisen regarding their potential impact on health. Recent research has suggested associations between the intake of artificial sweeteners and metabolic syndrome (16, 17), diabetes, and cardiovascular diseases (18). Specific links have been found between saccharin with migraines (19, 20) and insulin insensitivity (21), and between aspartame and prostate cancer and breast cancer (22). However, studies on the risk of CKD in relation to the consumption of artificial sweeteners still present conflicting findings (23). Some studies have reported an association between CKD and the intake of artificial sweeteners. In contrast, many others indicate no association between CKD or the progression of CKD and the consumption of artificial sweeteners. Therefore, existing research on artificial sweeteners in relation to CKD yields inconsistent and controversial results (24), raising the increasingly important question of their safety and reliability.

In observational studies, it is not possible to completely eliminate residual confounding, confounding factors, and reverse causality. Mendelian randomization (MR) analysis, however, overcomes the limitations of observational studies by using genetic variations associated with the exposure as instrumental variables to assess the correlation between the exposure and outcome (25). Compared to traditional epidemiological research, MR studies are less susceptible to residual confounding, confounding factors, and reverse causality, reducing biases in epidemiological research. While randomized controlled trials (RCTs) are considered helpful in determining causality (26), they are often constrained by cost, time, and ethical considerations, depending on the exposure characteristics and the disease under investigation. Furthermore, MR analysis may provide more convincing evidence than traditional observational studies and offer new insights into the treatment and diagnosis of diseases.

Therefore, considering all these factors, our study aimed to address this uncertainty by investigating the association between the intake of artificial sweeteners in the population, evaluating kidney function biochemical indicators, and their relationship with CKD. We utilized data from the National Health and Nutrition Examination Survey (NHANES) and employed MR analysis to determine the relationship between the intake of artificial sweeteners and impaired kidney function.

## 2 Materials and methods

### 2.1 NHANES

#### 2.1.1 The study population in NHANES

NHANES is a series of cross-sectional, complex, multi-stage surveys conducted by the National Center for Health Statistics (NCHS) in the United States. It utilizes a complex, multi-stage probability sampling procedure to ensure the representation of participants from different geographical locations and sufficient racial/ethnic diversity. The purpose of NHANES is to assess the health and nutritional status of the civilian, non-institutionalized population in the United States (27). The survey collects data from approximately 5,000 individuals each year through interviews, physical examinations, and laboratory tests. The NHANES research protocol has been approved by the Ethics Review Board of the National Center for Health Statistics, and all procedures are conducted in accordance with relevant approved guidelines and regulations, with informed consent obtained from each participant. The specific analytical methods can be found in the NHANES Laboratory and Medical Technologists Procedures Manual. Our current study combined two survey cycles (2003–2006). A total of 20,470 study participants from the 2003–2006 NHANES cycles were included. These two cycles included participants with information on kidney function and the Food Frequency Questionnaire (FFQ_C).

**Figure 1:**
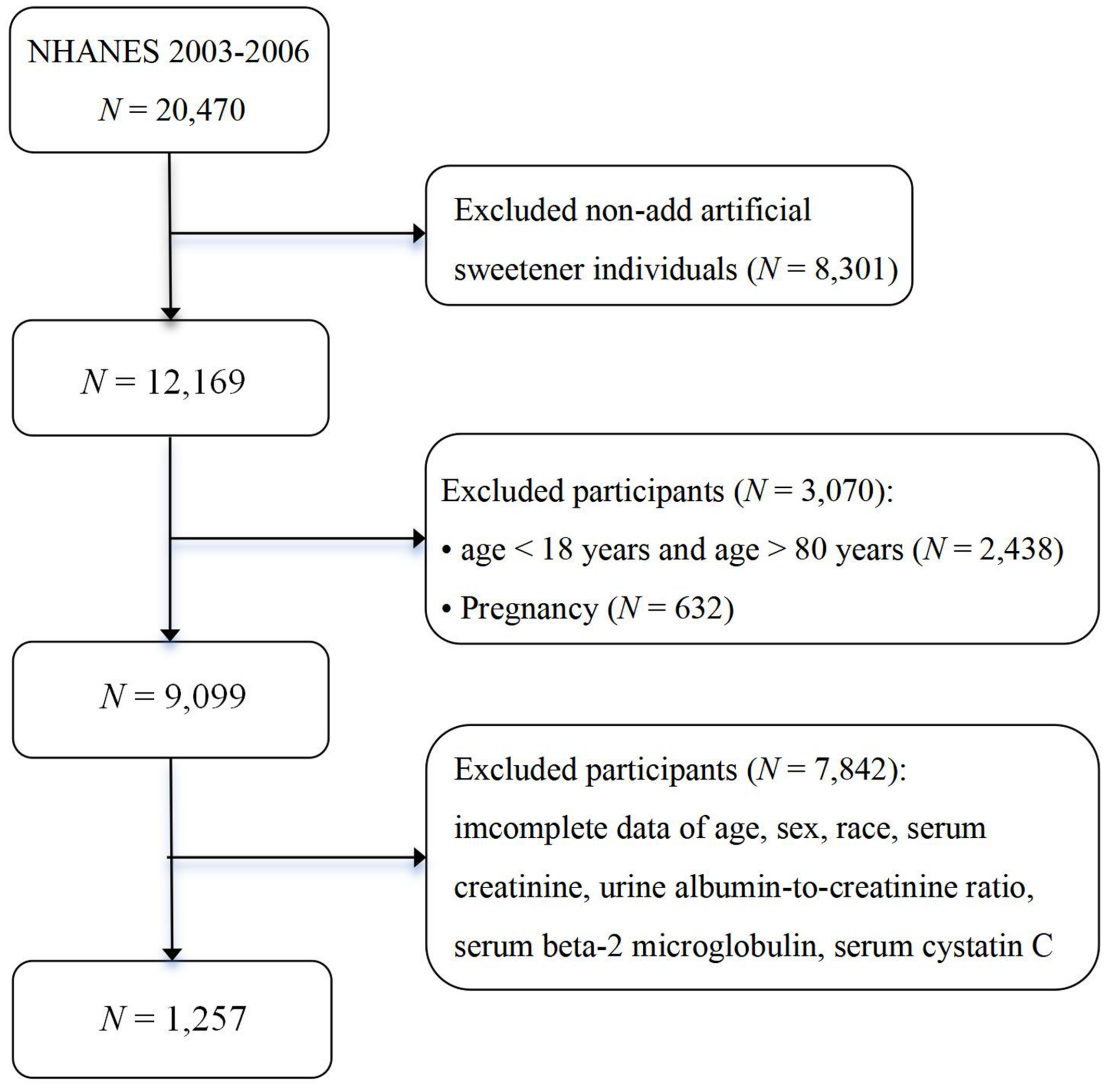
The flowchart of this study.

In the NHANES surveys conducted in 2003–2004 and 2005–2006, 6,306 and 5,863 participants, respectively, responded to questions regarding the use of artificial sweeteners. After excluding 5,763 participants from 2003 to 2004 and 5,149 participants from 2005 to 2006 who lacked relevant measurement indicators, biochemical markers, and associated weights, the final analytical sample comprised 1,257 respondents from the National Health and Nutrition Examination Survey conducted from 2003 to 2006. The participants had complete anthropometric measurements and demographic characteristics data. The flowchart for this study is presented in Figure 1.

#### 2.1.2 Artificial sweetener consumption data collection

Dietary data in NHANES is collected through individual 24-h dietary recall interviews conducted by the Mobile Examination Center (MEC). The assessment of dietary intake is based on 24-h recalls obtained by trained interviewers during the participant’s visit to the MEC. The interviews are conducted using a computer-assisted dietary interview system with standardized probes, namely the Automated Multiple-Pass Method (AMPM) developed by the United States Department of Agriculture. The AMPM collects information on the types and quantities of all food and beverages consumed in the 24 h preceding the dietary interview. The AMPM is designed to ensure more comprehensive and accurate data collection while reducing the burden on participants. The data on the intake of artificial sweeteners in the diet is reported in the FFQ_C, which includes two 24-h dietary recall interviews and information on dietary supplement use, food security, and dietary behaviors obtained through the dietary questionnaire. The FFQ_C collects information on the frequency of food consumption over the past 12 months. All participants were asked whether artificial sweeteners were added to their diet. Our study includes complete FFQ_C and covariate data.

#### 2.1.3 Measurement of indicators of renal function

Detailed descriptions of biochemical analysis methods can be found in the NHANES Laboratory/Medical Technologists Procedures Manual. Renal function indicators are analyzed using the Beckman Synchron LX20, with the LX20 modular chemistry side employing the Jaffe rate method (kinetic alkaline picrate) to determine the creatinine (Cr) concentration in serum, plasma, or urine. The calibration of creatinine is traceable to the isotope dilution mass spectrometry reference method. Urinary creatinine and urinary albumin (evaluated using random urine samples with solid-phase fluorescence immunoassay) are used to calculate the urine albumin-to-creatinine ratio (UACR). Serum beta-2 microglobulin (β2M) and cystatin C (CYSC) are measured using immunoturbidimetric and immunonephelometric assays on the automated multichannel analyzer Siemens Dimension Vista 1,500.

The estimated glomerular filtration rate (eGFR) is the gold standard for assessing renal function. Traditionally, equations based on creatinine have been used to calculate the estimated glomerular filtration rate. However, the secretion of Cr in the renal tubules can systematically overestimate GFR and be influenced by factors other than renal function, such as low muscle mass, low activity level, vegetarian diet, frailty, lower limb amputation, end-stage heart failure or liver failure, and the use of drugs that inhibit proximal tubular secretion of Cr. In 2021, the American Kidney Society workgroup recommended using CYSC to assess GFR in adults with or at risk for CKD. Although CYSC is not influenced by muscle mass or diet, factors such as obesity, hypothyroidism, smoking, and systemic corticosteroid use were positively associated with CYSC values. Therefore, the equation for eGFRcr-cys based on Cr and CYSC can provide the most accurate eGFR values. The calculation equation is as follows: eGFR (mL/min/1.73 m^2^) = 135 × min (Bcr/κ, 1)^α^ × max (Bcr/κ,1)^−0.601^ × min (Bcysc/0.8, 1)^−0.375^ × max (Bcysc/0.8, 1)^−0.711^ × 0.995^age^[×0.969 if female] × 1[1.08 if black] where Bcr is blood Cr, Bcyst is blood CYSC, κ is 0.7 for females and 0.9 for males, α is −0.248 for females and − 0.207 for males, min indicates the minimum of Bcr/κ or 1, and max indicates the maximum of Bcr/κ or 1 (28).

#### 2.1.4 Primary variables and covariables

In this study, participants were categorized into groups based on the frequency of artificial sweetener consumption reported in FFQ_C: never added (never), occasional (less than once a month, 1–3 times a month, once a week), regular (2–4 times a week, 5–6 times a week, once daily), and frequent (2–3 times daily, 4–5 times daily, 6 times or more daily). The renal function indicators included eGFR, UACR, CYSC, β2M, and serum creatinine (SCR). Blood pressure measurements were taken three times for all eligible individuals using a mercury sphygmomanometer at the MEC, including systolic blood pressure (SBP) and diastolic blood pressure (DBP). The average of the three blood pressure measurements, including SBP and DBP, was analyzed. Hypertension (HTN) was defined as a self-reported history of hypertension or SBP ≥140 mmHg or DBP ≥90 mmHg. CKD was defined as a self-reported history of CKD. Diabetes mellitus (DM) was a self-reported history of diabetes or fasting blood glucose ≥7.0 mmol/L. Gender was divided into male and female. Race/ethnicity was categorized as Mexican American, Other Hispanic, non-Hispanic white, non-Hispanic black, and other races (including multiple races). Education level was divided into less than 9th grade, 9th-11th grade (including 12th grade without a diploma), high school graduate, and college and above. The poverty income ratio (PIR) was expressed as the ratio of family income to the poverty line, with PIR values less than 1 indicating income below the poverty line and values greater than 1 indicating income above the poverty line. Self-reported smoking of less than 100 cigarettes was defined as a nonsmoker, while 100 or more cigarettes were defined as a smoker. Self-reported alcohol consumption of less than 12 drinks per year was defined as nondrinker. Body mass index (BMI, kg/m^2^) was calculated as weight (kg) divided by height squared (m^2^). All analysis models were adjusted for the following covariates: age, gender, race/ethnicity, BMI, education level, PIR, smoking status, alcohol consumption, fasting blood glucose, HTN, DM, CKD, triglycerides (TG), and high-density lipoprotein cholesterol (HDL).

#### 2.1.5 Statistic analysis

In this study using NHANES data, mean and standard error of the mean (SEM) were used to compare groups for continuous variables using one-way analysis of variance (ANOVA). In contrast, percentages were used for comparing groups of categorical variables using chi-square tests. To assess the normality of the data, the Kolmogorov–Smirnov test, and variables deviating from a normal distribution were log-transformed. Consequently, all renal function markers (UACR, eGFR, CYSC, SCR, β2M) were log-transformed to achieve a normal distribution. Adjusted means for renal function markers (UACR, eGFR, SCR, CYSC, β2M) in artificial sweetener groups were evaluated using ANCOVA. These models were adjusted for age, gender, race, BMI, education level, PIR, smoking status, alcohol consumption, fasting blood glucose, HTN, DM, TG, and HDL. Logistic regression models were conducted with similar adjustment strategies, using three different levels of adjustment: model 1 (age, gender, race, BMI, education level, PIR), model 2 (age, gender, race, BMI, education level, PIR, smoking status, alcohol consumption, fasting blood glucose, HTN), and model 3 (age, gender, race, BMI, education level, PIR, smoking status, alcohol consumption, fasting blood glucose, HTN, DM, TG, HDL). ORs and 95% confidence intervals (CIs) for renal function markers associated with artificial sweetener groups were derived. The reference value was always the group that never added artificial sweeteners. Trend tests were conducted by treating artificial sweetener intake groups as categorical variables. Taking into account the masking variance, clustered design, and differential sampling probabilities, the sample weights provided by NHANES were applied in this observational study. Four-year dietary independent weights were used to assess all samples, and data analysis followed CDC guidelines for analyzing complex NHANES data. All statistical analyses were conducted using SPSS software version 26.0, and two-sided *p*-values less than 0.05 were considered statistically significant.

### 2.2 Mendelian randomization

#### 2.2.1 Research design

**Figure 2:**
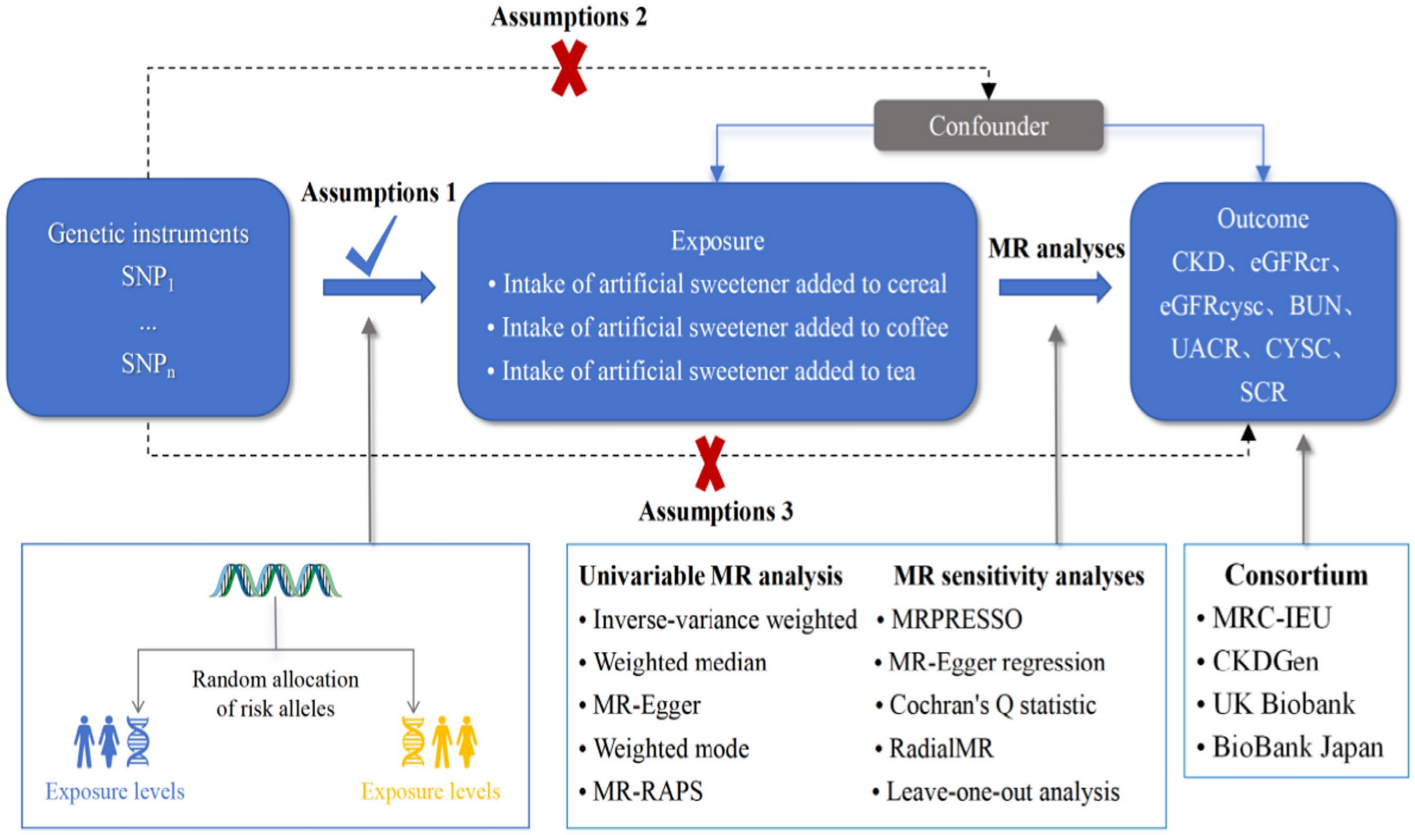
MR assumptions and study design.

A Mendelian randomization analysis was employed as a causal inference approach to assess the association between artificial sweeteners and CKD as well as renal function. Single-nucleotide polymorphisms (SNPs) were used as instrumental variables, and summary statistics data from various genome-wide association studies (GWAS) were analyzed to evaluate the effect of exposure on the outcomes. Essentially, we analyzed summary statistics data for exposure (artificial sweeteners) and outcomes (renal function markers and CKD) from different GWAS to assess the impact of the former on the latter. Figure 2 provides an overview of the design and hypotheses of the MR study. This study was conducted following MR-STROBE guidelines to enhance the reporting of observational epidemiological studies.

#### 2.2.2 Genetic predictors of exposure

The GWAS datasets related to artificial sweeteners were derived from the UK Biobank. Three European population GWAS datasets were compiled, including GWAS datasets for artificial sweetener intake in cereals, tea, and coffee. The combined datasets included 64,949 individuals and comprised 9,851,867 SNP loci. The genetic data mentioned can be downloaded from the MRC-IEU Open GWAS Project database, available at https://gwas.mrcieu.ac.uk/.

#### 2.2.3 Genetic predictors of outcome

The GWAS dataset related to CKD was obtained from the FinnGen Biobank, which studied 500,000 individuals of Finnish descent and enriched the data on rare and late-onset diseases. The project included 1,932 clinical endpoints GWAS, identifying genome-wide significant associations at 2,491 loci, including 148 putative causal coding variants, with 62 of them being over twice enriched in the Finnish population and having a low minor allele frequency (<10%). This study retrieved summary statistics data for CKD in European populations, including 3,902 cases and 212,841 controls. The definition of CKD in this study strictly followed the GB61 classification in ICD11 (CKD Stage I: GB61.0; CKD Stage II: GB61.1; CKD Stage IIIa: GB61.2; CKD Stage IIIb: GB61.3; CKD Stage IV: GB61.4; CKD Stage V: GB61.5). Summary statistics data for eGFR were sourced from the GWAS summary statistics for eGFRcrea in the CKDGen Consortium (*n* = 765,348, predominantly of European ancestry), and the GWAS results for eGFRcrea generated in the UK Biobank (UKB) (Application Number 20272, *n* = 436,561, of European ancestry), with adjustments for age, sex, and other study-specific covariates. The summary statistics data for SCR and blood urea nitrogen (BUN) were obtained from a publicly available GWAS dataset, which involved a deep phenotyping GWAS (diseases, biomarkers, and drug use) in the Japan Biobank (*n* = 179,000) and a GWAS meta-analysis conducted in the UK Biobank and FinnGen (combined *n* = 628,000), with adjustments for age, age squared, sex, age × sex, age squared × sex, and the first 20 principal components as covariates. The summary statistics data for CYSC were collected and provided by the UKB, with the primary analysis restricted to individuals reporting European ancestry (459,327 participants, representing 94% of the cohort) and further limited to a genetically homogeneous and unrelated subset of 337,539 White British participants, excluding a small number of participants who withdrew from the UKB (149 individuals). The summary statistics data for UACR were derived from data on unrelated proteinuria patients of European ancestry (382,500 individuals) in the UKB, with exclusions based on gender mismatch, absence of reported relationships, gender chromosome aneuploidies, and excessive heterozygosity or missingness. The diagnosis of CKD was based on recognized histological and radiological criteria. Recommended diagnostic criteria include albuminuria [UACR ≥30 mg/g] and persistent reduction in kidney function [eGFR <60 mL·min^−1^·1.73 m^−2^] for a duration of more than 3 months. The GWAS summary data used for renal function markers in this study were obtained from large-scale GWAS meta-analyses, including the DIAbetes Genetics Replication and Meta-analysis (DIAGRAM), UKB, BioBank Japan (BBJ), and FinnGen BioBank. The genetic information in the GWAS datasets pertaining to artificial sweeteners, CKD, and renal function markers (including UACR, eGFRcr, eGFRcysc, CYSC, SCR, and BUN) is summarized in Table 1.

**Table 1:** Summary of GWAS genetic data information.

	Characteristics	GWAS ID	Unit	Consortium	Published year	Sample size	Number of SNPs	PMID or trait
Exposure	Artificial sweetener	ukb-b-3143	SD unit	MRC-IEU	2018	64,949	9,851,867	Trait: intake of artificial sweetener added to cereal
		ukb-b-5867	SD unit	MRC-IEU	2018	64,949	9,851,867	Trait: intake of artificial sweetener added to tea
		ukb-b-1338	SD unit	MRC-IEU	2018	64,949	9,851,867	Trait: intake of artificial sweetener added to coffee
Outcome	CKD	finn-b-N14_CHRONKIDNEYDIS	NA	FinnGen	2021	25,186	16,380,459	Trait: chronic kidney disease
	eGFRcr	ebi-a-GCST90103634	ml/min/1.73 m2	CKDGen, UK Biobank	2021	1,004,040	8,168,311	PMID: 34272381
	eGFRcysc	ebi-a-GCST90103635	ml/min/1.73 m2	CKDGen, UK Biobank	2021	460,826	7,250,211	PMID: 34272381
	SCR	ebi-a-GCST90018979	μmoI/L	CKDGen, UK Biobank, BBJ	2021	344,104	19,034,241	PMID: 34594039
	CYSC	ebi-a-GCST90025945	mg/L	UK Biobank	2021	437,846	4,232,088	PMID: 34226706
	UACR	ebi-a-GCST006586	mg/g	UK Biobank	2018	382,500	11,684,850	PMID: 30220432
	BUN	ebi-a-GCST90018948	mmol/L	CKDGen, UK Biobank, BBJ	2021	344,052	19,049,084	PMID: 34594039

CKD, chronic kidney disease; eGFRcr, based on creatinine estimated glomerular filtration rate; eGFRcysc, based on cystatin C estimated glomerular filtration rate; SCR, Serum creatinine; CYSC, Serum cyscatin C; UACR, Urinary albumin creatinine ratio; BUN, blood urea nitrogen, NA, Not Available; SNP, single nucleotide polymorphism, BBJ, BioBank Japan; CKDGen, Chronic Kidney Disease Genetics Consortium.

#### 2.2.4 Instrumental variable filtering

The MR analysis relies on genetic instrumental variables that must satisfy three fundamental assumptions: (1) the assumption of association, indicating a close intertwining between the instrumental variables and the exposure factor; (2) the assumption of independence, stating that the instrumental variables are detached from any potential confounding factors that might impact the exposure-outcome relationship; and (3) the assumption of exclusivity, asserting that the instrumental variables influence the outcome solely through their effect on the exposure. Only when these three core assumptions are fulfilled can the MR study effectively counterbalance latent sources of confounding. Based on the initial assumption in MR analysis, this study proposes selecting instrumental variables as SNPs from the GWAS dataset of the exposure variable. These SNPs should display significant genomic associations (*p* < 5 × 10^−8^), exhibit no linkage disequilibrium (LD) (*r*^2^ < 0.001 within a 1 Mb distance), and possess a minimum minor allele frequency (MAF) greater than 1%. Unfortunately, it is unfortunate that a limited number of SNPs significantly associated with artificial sweetener intake in relation to CKD and renal function markers meet the *p* < 5 × 10^−8^ criterion. To further delve into the connection between artificial sweetener intake and CKD, as well as renal function markers, and obtain more comprehensive findings, the genetic instrument association threshold is thus adjusted to *p* < 1 × 10^−5^ (29). After identifying each corresponding significant SNP, LD analysis is carried out using the European genotype data from the 1,000 Genomes Project as the reference template. Only the SNP with the lower *p*-value is retained from the SNPs displaying LD (R^2^) above the specified threshold (*R*^2^ = 0.001). When extracting information on the instrumental variables from the outcome variable dataset, palindrome SNPs exhibiting inconsistent allele frequencies between the exposure and outcome datasets are removed. For missing SNPs in the exposure and outcome variable datasets, proxy SNPs with strong LD (*R*^2^ > 0.8) are utilized as substitutes. The effects on the outcome, along with the corresponding allele values, are reported for the proxy SNP and the target SNP. The association assumption is evaluated using the F-statistic. Typically, an instrumental variable is considered strong when the F-statistic exceeds 10, calculated as F = (R^2^ × (N – K − 1))/((1 − R^2^) × k), where N represents the sample size of the exposure database and K represents the number of SNPs, while R^2^ denotes the proportion of variance explained by the SNPs in the exposure database. For fewer than 10 instrumental variables, the formula for calculating R^2^ is R^2^ = 2 × EAF × (1 − EAF) × β^2^, where EAF signifies the effect allele frequency, and β is the allele effect. For 10 or more instrumental variables, the formula for calculating R^2^ is R^2^ = (2 × EAF × (1 − EAF) × β^2^)/[(2 × EAF × (1 − EAF) × β^2^) + (2 × EAF × (1 − EAF) × N × SE(β)^2^)] (30), where SE represents the standard error of the β estimate. Blindly excluding SNPs with *F* < 10 could introduce further biases. A search for these SNPs related to artificial sweetener intake in the PhenoScanner database is conducted, while excluding known SNPs associated with CKD risk factors (including BMI, diabetes, hypertension, heart failure, smoking, alcohol consumption, and lipid-related SNPs). The selected instrumental variables should include information regarding their reference alleles, effect alleles, effect allele frequencies, effect sizes (beta), standard errors (SE), and *p*-values from both the exposure and outcome dataset. To accommodate the second and third assumptions, we employed the MR-Egger method, with a horizontal pleiotropy test conducted based on the intercept and its corresponding *p*-value from MR-Egger regression.

#### 2.2.5 Statistical analyses

Our study aimed to assess the genetic predictive impact of artificial sweetener intake on CKD and various renal function-related indicators (eGFRcr, eGFRcysc, BUN, UACR, CYSC, and SCR). CKD serves as the primary outcome, while the secondary outcomes encompass different renal function indices. The genetic data selected for analysis exclusively come from European populations to mitigate the biases resulting from population stratification. Four methods are employed in this study to evaluate the causal relationship between artificial sweetener intake and renal function markers, as well as the risk of CKD: inverse-variance weighted method (IVW), weighted median estimator (WME), MR-Egger regression, and mode-based estimation (simple and weighted modes). The IVW method assumes that all SNPs serve as valid genetic instruments, assuming no horizontal pleiotropy. The WME method allows for the presence of horizontal pleiotropy in the instrumental variables, but requires that over 50% of the SNPs function as valid instruments. The MR-Egger method permits causal inference even when all SNPs are considered invalid instruments. Given the consideration for potential weak instrumental variables in the analysis, we employed the recently proposed MR-RAPS method to enhance the reliability of our results. MR-RAPS enables robust adjustment for pleiotropy using a robust adjusted profile score and provides reliable inferences when conducting MR analysis with a large number of weak instrumental variables, particularly when both the exposure and outcome variables are complex (31).

#### 2.2.6 Sensitivity analysis

To satisfy the second and third assumptions of MR analysis, tests for instrument heterogeneity and outliers are conducted using Cochran’s Q test (32) and the MR-PRESSO (33) (Mendelian Randomization Pleiotropy RESidual Sum and Outlier) global test. Cochran’s Q test is employed to assess heterogeneity in the IVW model, with a significance threshold of *p* < 0.05 indicating the presence of heterogeneity. A random-effects model is utilized for causal inference within the IVW framework in such cases. In the MR-PRESSO analysis, not only can the magnitude of instrument pleiotropy be estimated, but the effect size between the exposure and outcome variables can also be calculated after removing potential outliers, thereby allowing for an assessment of the results before and after correction. Sensitivity analysis is performed using a leave-one-out approach, where each instrumental variable SNP is excluded in turn, and the remaining SNPs are used to assess causal associations through the IVW method. The impact of each SNP on the MR analysis is determined by observing whether the effect estimate crosses the null value with a significance threshold of α = 0.05. Causal estimates are presented as ORs along with their corresponding 95% CIs. To enhance the visualization of IVW results, we utilized a radial plot and the RadialMR approach. RadialMR eliminates the need for genetic data re-encoding and allows for direct detection of outliers or potential pleiotropic SNPs, thereby providing more reliable causal estimates (34). Considering multiple testing, the two-sided *p*-values are adjusted using the Benjamini-Hochberg method to control the false discovery rate (FDR), a widely used approach for multiple hypothesis testing correction. The P.adjust function from the stats package (v4.2.3) is employed to control the FDR at a level of 5%. Adjusted *p*-values <0.05 are considered statistically significant. All statistical analyses are performed using R version 4.2.3 and the packages “MendelianRandomization,” “MR-PRESSO,” “TwoSampleMR,” “mr-raps,” and “RadialMR.”

#### 2.2.7 Ethics

The data utilized in this study were derived entirely from published research or publicly available GWAS summary data. As the study did not involve the collection of original data, ethical approval from an ethics committee was not required. Ethical approval for the publicly available summary statistics data was previously obtained and documented in the original GWAS publications. Each study included in our analysis received approval from its respective institutional ethics review board, and all participants provided written informed consent.

## 3 Results

### 3.1 Association between artificial sweetener intake and indicators of CKD and renal function in NHANES

A total of 1,257 NHANES participants met the inclusion criteria for the analysis, and 3.1% of the patients were found to have CKD. Table 2 presents the baseline characteristics of the participants divided into CKD and non-CKD groups. Overall, 53.9% of the participants were female, and there was no significant difference in gender between CKD and non-CKD groups (*p* = 0.052). Compared to those without CKD, participants with CKD were more likely to be non-Hispanic Black (28.2% vs. 17.1%), Mexican American (20.5% vs. 18.7%), and of other races (5.1% vs. 3.4%), with no significant differences in the distribution of CKD among different racial groups (*p* = 0.300). Participants with CKD were more likely to have lower levels of education compared to those without CKD, with a higher percentage having education levels below 9th grade (25.6% vs. 10.9%), 9-11th grade (17.9% vs. 14.8%), high school graduation (17.9% vs. 25.4%), and college graduation or higher (7.7% vs. 22.1%). These differences in education levels between groups were statistically significant (*p* = 0.017). The mean age of the overall population was 52.17 years, and participants with CKD were older than those without CKD (58.82 vs. 51.96 years, *p* = 0.024). Patients with CKD had higher BMI (*p* = 0.037), higher triglyceride levels (*p* = 0.163), higher poverty-income ratio (2.15 vs. 2.80, *p* = 0.012), higher fasting blood glucose (*p* < 0.001), and a higher likelihood of having diabetes (35.9% vs. 5.3%, *p* < 0.001) and hypertension (61.5% vs. 37.0%, *p* = 0.002) compared to those without CKD (see Table 2).

**Table 2:** Demographic characteristics of subjects for the whole sample and stratified by CKD status.

Characteristics		Total (*n* = 1,257)	With CKD (*n* = 39)	Without CKD (*n* = 1,218)	*p*-value
Sex (*n*, %)	Men	579 (46.1)	12 (30.8)	567 (46.6)	0.052
	Women	678 (53.9)	27 (69.2)	651 (53.4)	
Age, years (mean, se)		52.17 ± 0.53	58.82 ± 2.53	51.96 ± 0.54	0.024
Race/ ethnicity (*n*, %)	Mexican American	236 (18.8)	8 (20.5)	228 (18.7)	0.300
	Other Hispanic	25 (2.0)	0 (0.0)	25 (2.1)	
	Non-Hispanic White	733 (58.3)	18 (46.2)	715 (58.7)	
	Non-Hispanic Black	219 (17.4)	11 (28.2)	208 (17.1)	
	Other races including multiracial	44 (3.5)	2 (5.1)	42 (3.4)	
Education level (*n*, %)	Less than 9th Grade	143 (11.4)	10 (25.6)	133 (10.9)	0.017
	9th-11th Grade (Includes 12th grade with no diploma)	187 (14.9)	7 (17.9)	180 (14.8)	
	High School Grad/GED or Equivalent	316 (25.1)	7 (17.9)	309 (25.4)	
	Some College or AA degree	339 (27.0)	12 (30.8)	327 (26.8)	
	College Graduate or above	272 (21.6)	3 (7.7)	269 (22.1)	
Income to poverty (mean, se)		2.78 ± 0.05	2.15 ± 0.22	2.80 ± 0.05	0.012
Hypertension (*n*, %)	Yes	475 (37.8)	24 (61.5)	451 (37.0)	0.002
	No	782 (62.2)	15 (38.5)	767 (63.0)	
Diabetes (*n*, %)	Yes	127 (10.1)	14 (35.9)	113 (9.3)	<0.001
	No	1,130 (89.9)	25 (64.1)	1,105 (90.7)	
Smoking (*n*, %)	Yes	635 (50.5)	21 (53.8)	614 (50.4)	0.673
	No	622(49.5)	18 (46.2)	604 (49.6)	
Alcohol drinking (*n*, %)	Yes	868 (69.1)	30 (76.9)	838 (68.8)	0.280
	No	389 (31.9)	9 (23.1)	380 (31.2)	
Body mass index (kg/m^2^) (mean, se)		28.85 ± 0.18	30.97 ± 1.30	28.78 ± 0.18	0.037
Serum triglycerides (mmol/L) (mean, se)		1.71 ± 0.04	1.99 ± 0.19	1.70 ± 0.04	0.163
Serum high density lipoprotein (mmol/L) (mean, se)		1.42 ± 0.01	1.44 ± 0.07	1.42 ± 0.01	0.667
Fasting blood glucose (mmol/L) (mean, se)		5.69 ± 0.05	7.89 ± 0.76	5.62 ± 0.05	<0.001

Value expressed as mean and se or percentage. CKD, chronic kidney disease.

Among the analyzed sample participants, 67.6% (*n* = 850) never added artificial sweeteners, 13.6% (*n* = 171) occasionally added artificial sweeteners, 11.6% (*n* = 146) frequently added artificial sweeteners, and 7.2% (*n* = 90) added artificial sweeteners regularly. Individuals who frequently added artificial sweeteners were older, predominantly female, non-Hispanic White, or Mexican American. They had higher household PIR and education levels. Additionally, they were more likely to have hypertension, diabetes, and other diseases. Specific baseline characteristics are provided in Table 3. In order to further investigate the impact of artificial sweeteners on kidney function in the suboptimal health population, individuals with an eGFR ranging from 60 to 89 mL/min/1.73 m^2^ were defined as suboptimal health. In this stage, the renal function of the population is mildly impaired, without apparent clinical symptoms. Therefore, subgroup analysis was conducted to explore the relationship between artificial sweetener intake and kidney function across various variables. Supplementary Table S1 reveals statistically significant differences (*p* < 0.05) in age, PIR, HDL, and fasting blood glucose among different groups of artificial sweetener consumption. However, no statistically significant differences were observed in the remaining variables.

**Table 3:** Baseline characteristics of the participants in the number of artificial-sweetener addition categories.

Characteristics		No addition (*n* = 850)	Occasional addition (*n* = 171)	Regular addition (*n* = 146)	Frequent addition (*n* = 90)	*p*-value
Sex (*n*, %)	Men	408 (48.0)	67 (39.2)	60 (41.1)	44 (48.9)	0.099
	Women	442 (52.0)	104 (60.8)	86 (58.9)	46 (51.1)	
Age, years (mean, se)		51.32 ± 0.66	50.32 ± 1.37	55.32 ± 1.36	58.53 ± 1.66	<0.001
Race/ ethnicity (*n*, %)	Mexican American	140 (16.5)	29 (17.0)	47 (32.2)	20 (22.2)	0.001
	Other Hispanic	21 (2.5)	0 (0.0)	2 (1.4)	2 (2.2)	
	Non-Hispanic White	515 (60.6)	99 (57.9)	65 (44.5)	54 (60.0)	
	Non-Hispanic Black	145 (17.1)	38 (22.2)	27 (18.5)	9 (10.0)	
	Other races including multiracial	29 (3.4)	5 (2.9)	5 (3.4)	5 (5.6)	
Education level (*n*, %)	Less than 9th Grade	87 (10.2)	14 (8.2)	26 (17.8)	16 (17.8)	0.010
	9-11th Grade (includes 12th grade with no diploma)	133 (15.6)	18 (10.5)	25 (17.1)	11 (12.2)	
	High School Grad/GED or Equivalent	224 (26.4)	46 (26.9)	31 (21.2)	15 (16.7)	
	Some College or AA degree	225 (26.5)	44 (25.7)	39 (26.7)	31 (34.4)	
	College Graduate or above	181 (21.3)	49 (28.7)	25 (17.1)	17 (18.9)	
Income to poverty (mean, se)		2.71 ± 0.05	3.17 ± 0.12	2.69 ± 0.13	2.83 ± 0.17	0.007
Hypertension (*n*, %)	Yes	283 (33.3)	76 (44.4)	71 (48.6)	45 (50.0)	<0.001
	No	567 (66.7)	95 (55.6)	75 (51.4)	45 (50.0)	
Diabetes (*n*, %)	Yes	48 (9.9)	23 (13.5)	32 (21.9)	24 (26.7)	<0.001
	No	802 (90.1)	148 (86.5)	114 (78.1)	66 (73.3)	
CKD (*n*, %)	Yes	18 (5.6)	9 (13.5)	7 (4.8)	5 (5.6)	0.035
	No	832 (94.4)	162 (86.5)	137 (95.2)	85 (94.4)	
Smoking (*n*, %)	Yes	438 (51.5)	80 (46.8)	67 (45.9)	50 (55.6)	0.325
	No	412 (48.5)	91 (53.2)	79 (54.1)	40 (44.4)	
Alcohol drinking (*n*, %)	Yes	587 (69.1)	116 (67.8)	99 (67.8)	66 (73.3)	0.802
	No	263 (30.9)	55 (32.2)	47 (32.2)	24 (26.7)	
Body mass index (kg/m^2^) (mean, se)		28.17 ± 0.22	30.53 ± 0.54	30.45 ± 0.55	29.45 ± 0.54	<0.001
Serum Triglycerides (mmol/L) (mean, se)		1.65 ± 0.04	1.74 ± 0.13	1.95 ± 0.14	1.81 ± 0.11	0.042
Serum High density lipoprotein (mmol/L) (mean, se)		1.42 ± 0.01	1.47 ± 0.03	1.36 ± 0.04	1.39 ± 0.04	0.145
Fasting blood glucose (mmol/L) (mean, se)		5.44 ± 0.05	5.94 ± 0.20	6.27 ± 0.19	6.64 ± 0.28	<0.001
Urinary albumin creatinine ratio (mg/g) (mean, se)		26.61 ± 5.88	41.53 ± 14.12	54.34 ± 18.28	74.22 ± 37.16	0.084
Serum creatinine (umol/L) (mean, se)		80.17 ± 0.79	78.27 ± 1.77	78.11 ± 1.85	81.72 ± 2.73	0.500
Serum Beta 2 Microglobulin (mg/L) (mean, se)		2.13 ± 0.03	2.15 ± 0.08	2.30 ± 0.12	2.32 ± 0.12	0.165
Serum cyscatin C (mg/L) (mean, se)		0.78 ± 0.01	0.78 ± 0.02	0.81 ± 0.02	0.86 ± 0.04	0.074
eGFRcr-cys (mL/min/1.73 m^2^) (mean, se)		65.34 ± 1.36	59.69 ± 3.08	56.08 ± 3.14	58.54 ± 4.34	0.021

Value expressed as mean and se or percentage. CKD, chronic kidney disease; eGFRcr-cys, based on creatinine and cystatin C estimated glomerular filtration rate.

Table 4 presents the results of unadjusted logistic regression analysis, indicating that compared to never adding artificial sweeteners, occasional artificial sweetener usage had an odds ratio (OR) of 2.568 (95% CI: 1.134–5.817, *p* = 0.024), regular usage had an OR of 2.328 (95% CI: 0.955–5.676, *p* = 0.063), and frequent addition had an OR of 2.719 (95% CI: 0.985–7.507, *p* = 0.054) (with a trend *p*-value of 0.009, as CKD was diagnosed using eGFR). However, after adjusting for age, gender, race, BMI, education level, PIR, smoking, alcohol consumption, fasting blood glucose, hypertension, diabetes, triglycerides, and HDL in a logistic regression model, the significance of the association between artificial sweetener usage and kidney function markers diminished. Adjusted mean levels of kidney function markers for different groups of artificial sweetener usage are presented in Table 5. Ln UACR remained non-significant and unchanged across the groups (0.945–0.953 mg/g, *p* = 0.956). Similarly, Ln β2M (0.702–0.685 mg/L, *p* = 0.476), Ln eGFR (3.916–3.975, *p* = 0.374), and Ln CYSC (0.265–0.279 mg/L, *p* = 0.808) showed no significant changes and maintained stability across the different artificial sweetener usage groups. However, Ln SCR showed a significant change (4.296–4.355 umol/L, *p* = 0.049), demonstrating statistical significance.

**Table 4:** Association between artificial sweetener consumption and clinically relevant renal chronic disease in adults.

Exposure	OR (95%CI), *p*-value			
	Crude	Model 1	Model 2	Model 3
Artificial sweetener group status				
No addition	1	1	1	1
Occasional addition	2.568 (1.134–5.817), 0.024	2.648 (1.142–6.137), 0.023	2.133 (0.879–5.175), 0.094	2.144 (0.883–5.208), 0.092
Regular addition	2.328 (0.955–5.676), 0.063	1.878 (0.747–4.721), 0.180	1.411 (0.547–3.643), 0.476	1.406 (0.544–3.630), 0.482
Frequent addition	2.719 (0.985–7.507), 0.054	2.320 (0.819–6.569), 0.113	1.499 (0.500–4.493), 0.470	1.503 (0.500–4.522), 0.468
*p* for trend	0.045	0.024	0.063	0.054

Crude: adjust for none.

Model 1: adjust for age, sex, race, BMI, education, and PIR.

Model 2: adjust for age, sex, race, BMI, education, PIR, smoking, drinking, fasting glucose, and HTN.

Model 3: adjust for age, sex, race, BMI, education, PIR, smoking, drinking, fasting blood glucose, HTN, DM, TG, and HDL.

*p*-values for trends were calculated by modeling rank values for artificial sweetener consumption as continuous variables. CI, confidence interval; OR, odds ratio.

**Table 5:** Adjusted (age, sex, race, education, PIR, HDL, alcohol consumption, smoking, BMI, HTN, TG, and DM) in the artificial sweetener groups imply levels of CKD indicators.

Characteristics	Artificial sweetener group status				*p*-value
	No addition	Occasional addition	Regular addition	Frequent addition	
Number of participants (*n*)	850	171	146	90	
Ln Urinary albumin creatinine ratio (mg/g)	0.945 ± 0.016	0.954 ± 0.035	0.927 ± 0.038	0.953 ± 0.048	0.956
Ln Serum creatinine (umol/L)	4.355 ± 0.008	4.340 ± 0.018	4.296 ± 0.020	4.324 ± 0.025	0.049
Ln Serum Beta 2 Microglobulin (mg/L)	0.702 ± 0.009	0.734 ± 0.021	0.707 ± 0.023	0.685 ± 0.029	0.476
Ln Serum cystatin C (Absolute value) (mg/L)	0.279 ± 0.008	0.265 ± 0.019	0.293 ± 0.020	0.279 ± 0.026	0.808
Ln eGFRcr-cys (mL/min/1.73 m^2^)	3.924 ± 0.011	3.916 ± 0.023	3.975 ± 0.029	3.929 ± 0.032	0.374

Values (from analysis of covariance) expressed as mean ± se. eGFRcr-cys, based on creatinine and cystatin C estimated glomerular filtration rate.

### 3.2 Causal relationship between artificial sweeteners intake and indicators of CKD and renal function in MR

In our study, we initially performed MR analysis using all the selected SNPs as instrumental variables (IVs). If the MR-PRESSO analysis detected significant levels of pleiotropy, we removed outliers (with *p*-values lower than the threshold in the MR-PRESSO outlier test) and performed MR analysis again. After removing the outliers through the MR-PRESSO outlier removal step conditional on the SNPs with *p*-values less than 1 in the MR-PRESSO outlier removal test. If there is still heterogeneity present, we used RadialMR to eliminate SNPs with outliers or potential pleiotropy. We should exercise caution in drawing conclusions if potentially influential SNPs were identified in the leave-one-out sensitivity analysis. Finally, since artificial sweeteners are consumed with other foods and beverages (added to cereals, tea, and coffee), it is challenging to attribute these findings solely to the intake of artificial sweeteners. Therefore, it is necessary to perform comparative analyses between the selected SNPs and SNPs significantly correlated with cereals, tea, and coffee, to exclude any common SNPs or SNPs that express phenotypes related to cereals, tea, and coffee.

#### 3.2.1 Impact of artificial sweetener intake on CKD

Table 6 presents the MR estimates for the causal relationship between adding artificial sweeteners in cereals, tea, and coffee and CKD. Using the IVW and MR-RAPS methods as primary analyses, no significant associations were found between the addition of artificial sweeteners in cereals, tea, and coffee and CKD in the European population. Specifically, no significant associations were observed for the addition of artificial sweeteners in cereals (IVW: OR = 1.37, β = 0.317, 95% CI = −0.571 to 1.204, *p* = 0.484; MR-RAPS: OR = 1.38, β = 0.325, 95% CI = −0.616 to 1.266, *p* = 0.499), tea (IVW: OR = 0.83, β = −0.185, 95% CI = −0.744 to 0.374, *p* = 0.517; MR-RAPS: OR = 0.83, β = −0.190, 95% CI = −0.779 to 0.398, *p* = 0.525), and coffee (IVW: OR = 1.17, β = 0.157, 95% CI = −0.390 to 0.703, *p* = 0.574; MR-RAPS: OR = 1.18, β = 0.163, 95% CI = −0.411 to 0.736, *p* = 0.579). The corrected *p*-values using the Benjamin-Hochberg method also did not indicate a causal relationship between adding artificial sweeteners in cereals, tea, and coffee and CKD (*p* > 0.05). The MR-Egger intercepts did not provide statistical evidence of directional pleiotropy (all *p*-values >0.05). The MR-PRESSO test did not identify potential pleiotropy among the included SNPs. However, the RadialMR plot suggested the presence of horizontal pleiotropy, indicating a potential influential SNP driving the causal relationship between exposure and outcome. Therefore, caution is warranted when removing all outliers since, even though classified as outliers, the SNPs should also be considered for their association with a separate phenotype representing a pleiotropic pathway of the outcome. The scatter plot demonstrates the estimated effects of SNPs on exposure (AS_cereal, AS_coffee, AS_tea) and CKD outcome (Supplementary Figure S4). The funnel plot indicates positions where directional pleiotropy exists for each outcome (Supplementary Figure S1). The leave-one-out analysis is depicted in Supplementary Figure S2, and the RadialMR plot is shown in Supplementary Figure S3.

**Table 6:** Association of genetically determined artificial sweeteners with CKD.

Outcome	Exposure	No. of SNPs used	F-statistic^*^	Mendelian randomization method	OR (95% CI)	*p*-value	Adjusted *p*-value^*^	Cochran’s Q (I^2^)	Q-value	MR-egger intercept (*p* value)	Outliers from MR-PRESSO	Outliers from MR-radial
CKD	Artificial sweetener added to cereal	29	91.60	Inverse-variance weighted	1.37 (0.57–3.33)	0.484	0.927	15.6 (0.00%)	0.971	0.001(0.934)	NA	rs72747173 rs9395027
				MR Egger method	1.29 (0.23–7.11)	0.773	0.927	15.6 (0.00%)	0.960			
				Weighted median	0.99 (0.27–3.61)	0.983	0.983					
				MR-RAPS	1.38 (0.54–3.54)	0.499	0.927					
	Artificial sweetener added to tea	28	223.29	Inverse-variance weighted	0.83 (0.47–1.45)	0.517	0.856	17.9 (0.00%)	0.906	−0.007(0.722)	NA	NA
				MR Egger method	1.02 (0.29–3.53)	0.977	0.977	17.8 (0.00%)	0.884			
				Weighted median	0.81 (0.37–1.76)	0.581	0.856					
				MR-RAPS	0.83 (0.46–1.49)	0.525	0.856					
	Artificial sweetener added to coffee	22	233.39	Inverse-variance weighted	1.17 (0.68–2.02)	0.574	0.579	20.5 (0.00%)	0.487	−0.021(0.213)	NA	rs11136419 rs59576512 rs78115985
				MR Egger method	2.04 (0.74–5.58)	0.181	0.579	18.9 (0.00%)	0.529			
				Weighted median	0.73 (0.34–1.58)	0.426	0.579					
				MR-RAPS	1.18 (0.66–2.09)	0.579	0.579					

MR-PRESSO, Mendelian randomization pleiotropy residual sum and outlier; CKD, chronic kidney disease; OR, odds ratio; CI, confidence interval; MR-RAPS, Robust adjusted profile score; adjusted *p*-value, The *p*-values are adjusted according to the Benjamin-Hochberg method; The mean F statistics of the instruments were calculated for IVW, WM, and MR-Egger. F-statistic*, The mean F statistics of the instruments were calculated for IVW, WM, and MR-Egger.

#### 3.2.2 Impact of artificial sweetener intake on relevant renal function indicators

**Figure 3:**
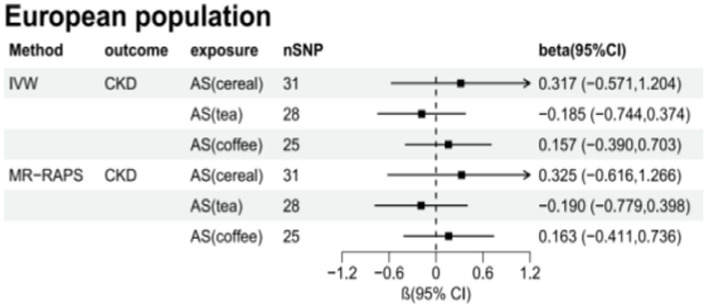
Total causal effects of artificial sweeteners on CKD in the European population.

**Figure 4:**
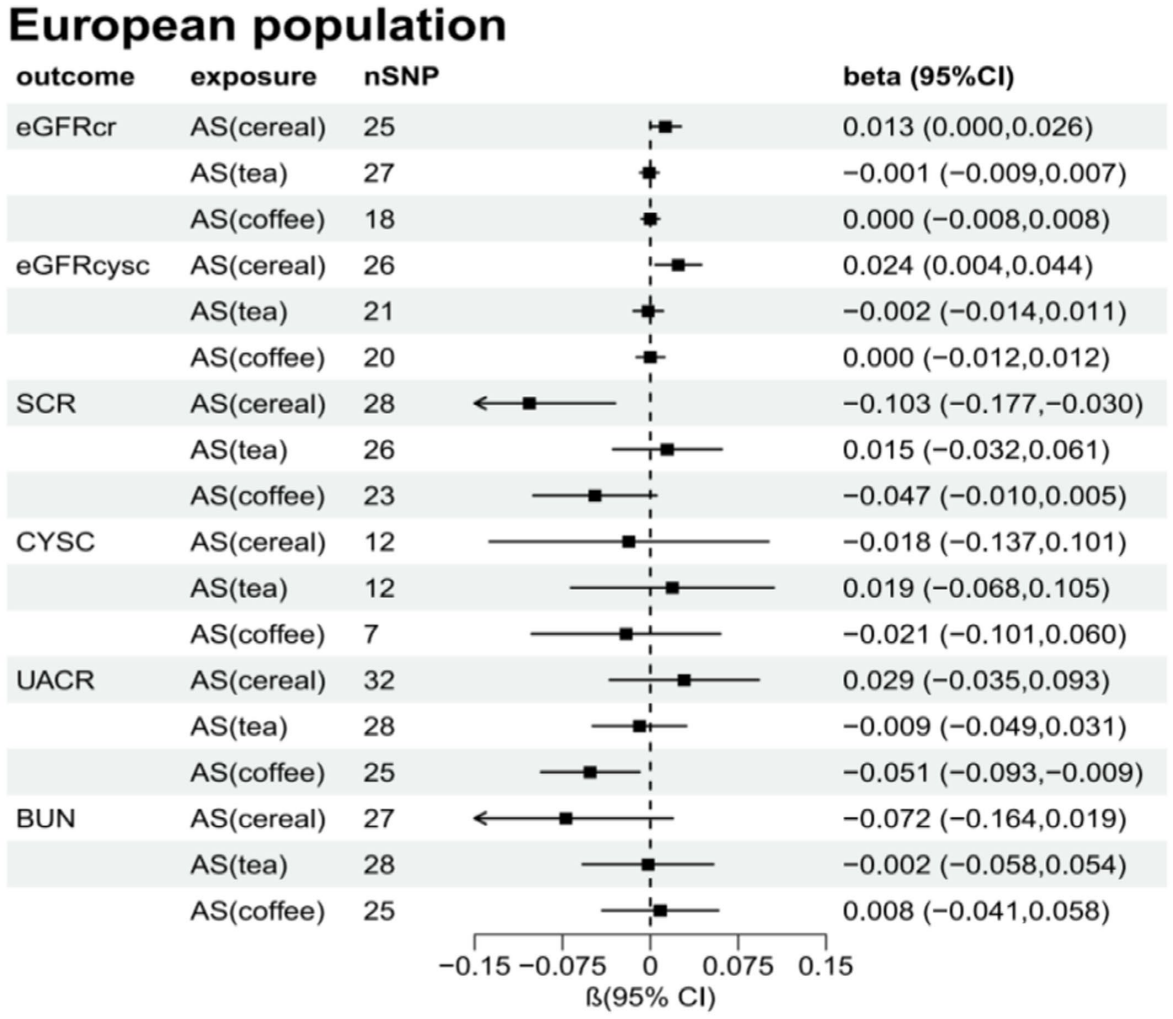
Total causal effect of artificial sweeteners on eGFRcr, eGFRcysc, BUN, UACR, CYSC, SCR in a European population (inverse-variance weighted method).

**Figure 5:**
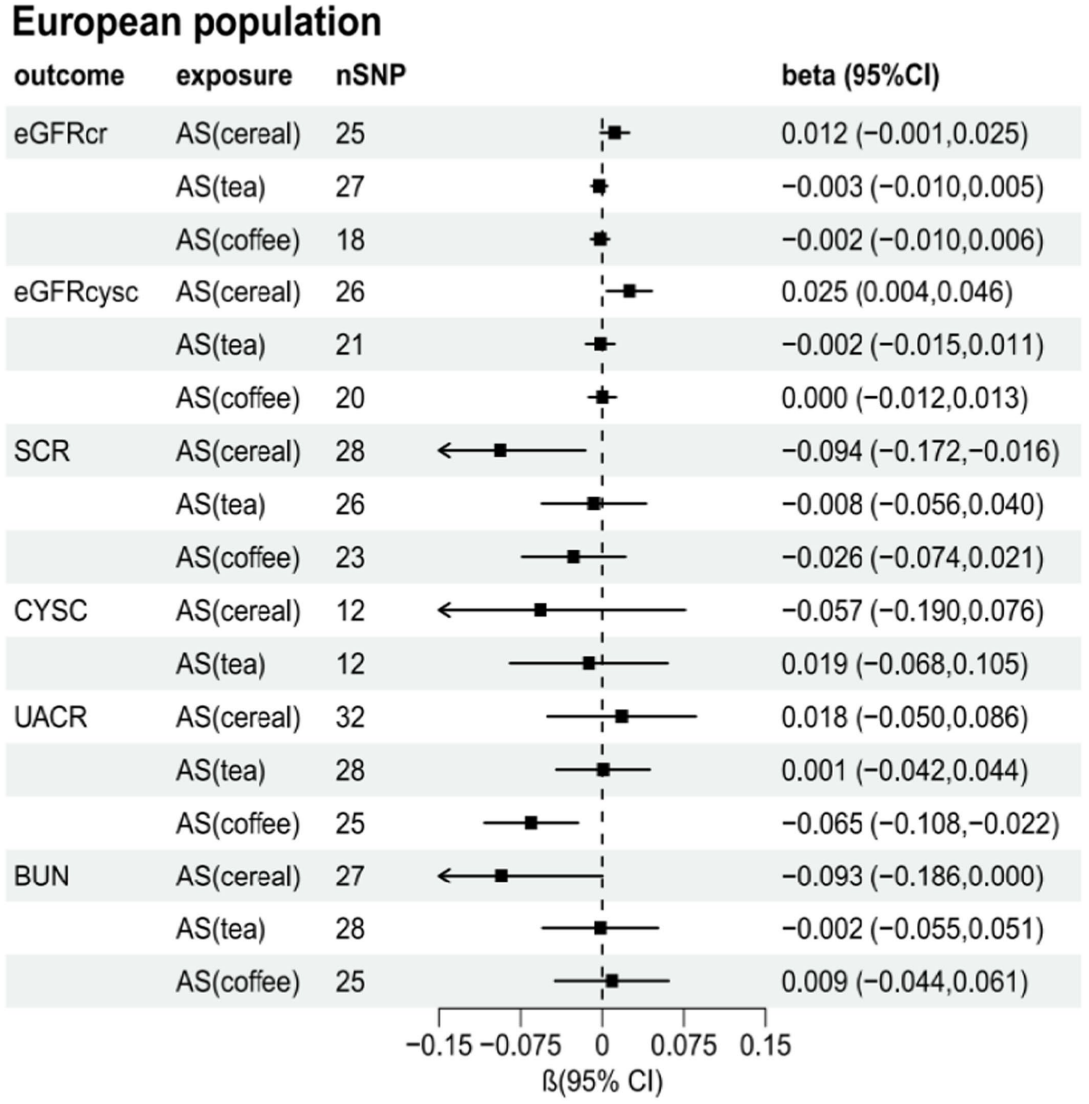
Total causal effect of artificial sweeteners on eGFRcr, eGFRcysc, BUN, UACR, CYSC, SCR in a European population (MR-RAPS method).

Supplementary Tables S2–S7 present the results of the MR analysis methods for the relationship between the addition of artificial sweeteners in cereals, tea, and coffee and renal function indicators. Using the IVW and MR-RAPS methods as primary analyses, we observed a positive correlation between the addition of artificial sweeteners in cereals (IVW: OR = 1.02, β = 0.024, 95% CI = 0.004 to 0.044, *p* = 0.017; MR-RAPS: OR = 1.03, β = 0.025, 95% CI = 0.004 to 0.046, *p* = 0.019) and eGFRcysc, as well as a negative correlation between the addition of artificial sweeteners in cereals (IVW: OR = 0.91, β = −0.091, 95% CI = −0.165 to −0.017, *p* = 0.017; MR-RAPS: OR = 0.91, β = −0.094, 95% CI = −0.172 to −0.016, *p* = 0.019) and SCR. However, after correction using the Benjamin-Hochberg method, the adjusted *p*-values were greater than 0.05 (adjusted *p*-value: 0.057 and 0.056), indicating suggestive evidence of potential correlations. Moreover, we found a negative correlation between the addition of artificial sweeteners in coffee (IVW: OR = 0.94, β = −0.063, 95% CI = −0.104 to −0.023, *p* = 0.002; MR-RAPS: OR = 0.94, β = −0.065, 95% CI = −0.108 to −0.022, *p* = 0.003) and UACR, and the adjusted *p*-value after Benjamin-Hochberg correction remained below 0.05 (adjusted *p*-value: 0.009). However, there was no causal relationship between adding artificial sweeteners in cereals, tea, and coffee and other renal function indicators (*p* > 0.05). Scatter plots (Supplementary Figures S4, S8, S12) were used to visualize the effect sizes of each MR analysis. MR-Egger, weighted median, MR-RAPS, and IVW estimates showed consistent results. Furthermore, sensitivity analyses using the MR-PRESSO test and RadialMR plot to remove potential pleiotropic SNPs indicated no evidence of pleiotropy or heterogeneity (Supplementary Tables S2–S7 and Supplementary Figures S3, S7, S11). Funnel plots depicted the balanced distribution of each SNP (Supplementary Figures S1, S5, S9). The leave-one-out analysis results demonstrated that excluding any single SNP in the genetic variation hardly biased the results (Supplementary Figures S2, S6, S10; see also Figures 3–5).

## 4 Discussion

This study represents the first comprehensive investigation of the relationship between artificial sweetener intake and CKD and renal function indicators using scale cross-sectional research and genetic data in an MR analysis framework. We examined the associations between artificial sweetener intake (added in cereals, tea, and coffee) and CKD and various renal function indicators, including UACR, eGFRcr, eGFRcysc, CYSC, SCR, and BUN. We explored their potential causal relationships. The results revealed that adding artificial sweeteners to coffee was associated with reducing UACR levels. We also observed associations between the addition of artificial sweeteners in cereals and eGFR and SCR. However, we did not find any genetic links between the addition of artificial sweeteners in cereals, tea, and coffee and CKD or other renal function indicators.

Previous studies on the relationship between artificial sweetener and UACR have yielded conflicting results (24). Cross-sectional studies found no association between artificial sweetener intake and UACR. However, other cross-sectional or cohort studies suggested that higher intake of artificial sweeteners may increase UACR. To date, there have been no reports of randomized controlled trials or MR studies investigating the impact of artificial sweetener intake on UACR. Hence, our contribution of evidence demonstrating the positive effects of artificial sweetener intake on UACR addresses a previously existing research gap. This beneficial effect may be attributed to its preventive effects on increased permeability induced by vascular endothelial growth factor (VEGF). Artificial sweeteners bind to the T1R2/T1R3 sweet taste receptors in G-protein-coupled receptors, leading to increased expression of vascular endothelial VE-cadherin, without affecting the Cyclic adenosine monophosphate (cAMP) or oxidative stress pathways in the glomerular microvascular system. Thus, artificial sweeteners protect the glomerular microvasculature from VEGF-induced barrier disruption in a sweet taste receptor-dependent manner (35). Furthermore, our study indicates a potential association between artificial sweetener intake and eGFR as well as SCR. Although previous case–control studies have suggested an improvement in eGFR and SCR with artificial sweetener intake, this association is unlikely to be causal. The improvement in eGFR and SCR may be attributed to a reduction in calorie intake. Specifically, the intake of artificial sweeteners as a replacement for high-calorie sugars reduces energy intake and improves body weight (36). This effect is particularly significant in obese individuals, as high BMI is directly associated with eGFR and SCR, and a reduction in BMI improves renal function. Further research is needed to investigate the impact of artificial sweetener intake on renal function in the general population.

Furthermore, our study suggests a potential correlation between artificial sweetener intake and eGFR and SCR. Although previous case–control studies have shown improvements in eGFR and SCR with artificial sweetener intake, this association is unlikely to be causal. The improvement in eGFR and SCR may be attributed to reduM Me in the diet. Specifically, the intake of artificial sweeteners replaces high-calorie sugars, resulting in reduced energy intake and improved weight. This effect is particularly significant in obese individuals, as high BMI is directly associated with eGFR and SCR, and reducing BMI can improve kidney function. The impact of artificial sweetener intake on kidney function in the general population requires further investigation.

Based on the previous short-term RCT results, we found no association between the consumption of artificial sweeteners and CKD. We observed no significant differences in kidney function indicators among the sub-healthy group regarding artificial sweetener intake. Similarly, a systematic review and meta-analysis has indicated that the consumption of artificially sweetened soda by CKD patients does not have any statistically significant impact on the risk of developing CKD (37). However, a long-term observational study has suggested an increased risk of CKD associated with long-term artificial sweetener intake, but this association seems to be limited to overweight or obese individuals, with no significant correlation observed in the general population (38). Nonetheless, our study did not discover any significant link between the consumption of artificial sweeteners and CKD. This could possibly be attributed to the heterogeneity in the diagnosis of CKD and the use of dichotomous variables rather than continuous variables, which may have resulted in insufficient statistical power (classifying a continuous variable into two categories can lead to a considerable loss of statistical power). Furthermore, our analysis of CKD was based on a combination of renal injury and glomerular filtration rate, suggesting that artificial sweeteners may play a role in protecting the integrity of the microvascular barrier, reducing glomerular vascular permeability, and thereby lowering the production of proteinuria, but they may not prevent other forms of kidney damage. The World Health Organization has issued guidelines (39) for non-diabetic populations, advising against the use of artificial sweeteners for weight control and the prevention of non-communicable diseases. Similarly, our results do not support the consumption of artificial sweeteners as a means to improve kidney function in CKD patients. Moreover, our results cast doubt on the consumption of artificial sweeteners in the general population as a means to maintain kidney function.

Nevertheless, despite diligent efforts to mitigate biases in our present study, we remain unable to definitively discern whether the observed effects on kidney function solely stem from artificial sweeteners or if they arise from the cumulative impact of artificial sweeteners in conjunction with other foods and beverages. The former cannot explain the effects of different types of artificial sweeteners on kidney function, while the latter may be due to the fact that the addition of artificial sweeteners makes foods and beverages (rich in dietary fiber, such as tea and coffee) more palatable, leading to increased consumption of these foods and beverages, which could have a protective effect. This is because, in comparison to artificial sweeteners, the intake of food and beverages constitutes the majority. Further research is necessary to unravel these findings, particularly considering the widespread use of artificial sweeteners in food and beverages and the increasing burden of kidney disease.

The primary strength of this study lies in the utilization of MR analysis combined with an observational study design using NHANES data. The large sample size of NHANES supports the inclusion of multiple factors as covariates in our multivariable-adjusted logistic regression analysis. Additionally, the use of MR analysis theoretically minimizes potential biases. Furthermore, the consistency between the results from MR analysis and the observational study enhances the reliability of the findings. In addition, we employed multiple methods to analyze heterogeneity and pleiotropy to ensure the robustness of our MR analysis. However, there are certainly limitations. For instance, the effects of artificial sweeteners can be influenced by various factors such as type, quantity (potentially multiple artificial sweeteners present), and concentration.

Additionally, we cannot ascertain potential interactions between different types of sweeteners or between sweeteners and other additives and ingredients. This study’s data on artificial sweeteners were derived from two 24-h dietary recall interviews in NHANES. Due to the retrospective nature of data collection, it may not precisely reflect individuals’ regular intake. To minimize biases, we excluded participants with only one 24-h recall interview. Recall bias may affect cross-sectional results, as dietary data in NHANES are self-reported. The inclusion of GWAS data from the UK Biobank for both the exposure and outcome in our MR analysis introduces the possibility of sample overlap, which could potentially compromise the reliability of the study’s findings. Furthermore, the cross-sectional and MR studies were not conducted on the same population, as our cross-sectional study utilized a multi-ethnic population in the United States. In contrast, the MR study focused on individuals of European ancestry. Future research should examine populations of the same ethnicity to control for potential confounding factors due to population heterogeneity and obtain more comprehensive results.

In conclusion, our study revealed the relationship between the intake of artificial sweeteners and kidney function. Genetic prediction showed a decrease in UACR levels when artificial sweeteners were added to coffee, but it had no impact on the risk of CKD. No association was found between genetic prediction of artificial sweetener intake and CKD or other kidney function measures. Although the estimated effect of artificial sweetener intake on UACR was relatively small and may lack clinical relevance, they could have significant implications for population-level health. The study results also question the protective role of artificial sweetener intake in the general population. While our study is comprehensive, aspects still warrant further exploration. Large-scale, multi-ethnic, and prospective studies are needed to address the limitations of the current research and provide more substantial evidence. As our understanding of the potential effects of artificial sweetener consumption on kidney function deepens, considering various factors such as the type of artificial sweeteners, interactions with other food components and additives, and studying specific populations (such as pregnant or lactating women, children, and individuals with diabetes) will be crucial in future research.

## 5 Conclusion

Our study does not substantiate a causal link between artificial sweetener consumption and CKD. Moreover, we identified a reduction in UACR levels only when artificial sweeteners were incorporated into coffee, albeit the effect being relatively modest and potentially lacking a substantial individual impact. Nonetheless, owing to pleiotropy and other inherent limitations in observational studies, there may be biases in the findings, warranting validation in more extensive MR studies. Consequently, our current research results do not provide any indication that artificial sweetener intake may elevate the risk of CKD.

## Data availability statement

The original contributions presented in the study are included in the article/Supplementary material, further inquiries can be directed to the corresponding author.

## Author contributions

ZR: Conceptualization, Data curation, Formal analysis, Investigation, Methodology, Software, Visualization, Writing – original draft, Writing – review & editing. YZhe: Conceptualization, Data curation, Formal analysis, Methodology, Writing – original draft. LY: Conceptualization, Data curation, Formal analysis, Methodology, Writing – original draft. YZha: Data curation, Formal analysis, Methodology, Writing – original draft. ZZ: Formal analysis, Methodology, Software, Writing – review & editing. HL: Formal analysis, Methodology, Software, Visualization, Writing – review & editing. XL: Formal analysis, Investigation, Methodology, Software, Writing – review & editing. JS: Investigation, Methodology, Software, Writing – review & editing. LZ: Investigation, Resources, Software, Writing – review & editing. RZ: Formal analysis, Resources, Software, Writing – review & editing. CL: Investigation, Methodology, Resources, Writing – review & editing. YG: Project administration, Resources, Supervision, Validation, Writing – review & editing. JG: Conceptualization, Data curation, Formal analysis, Investigation, Methodology, Resources, Software, Supervision, Validation, Writing – original draft, Writing – review & editing.

## Glossary

**Table:** 

ANOVA	Analysis of variance
AMPM	Automated multiple-pass method
AS	Artificial sweetener
β2M	Beta-2 microglobulin
BBJ	BioBank Japan
BUN	Blood urea nitrogen
BMI	Body mass index
CKD	Chronic kidney disease
CIs	Confidence intervals
Cr	Creatinine concentration
cAMP	Cyclic adenosine monophosphate
CYSC	Cystatin C
DIAGRAM	Diabetes genetics replication and meta-analysis
DM	Diabetes mellitus
DBP	Diastolic blood pressure
eGFR	estimated glomerular filtration rate
FDR	False discovery rate
FFQ_C	Food frequency questionnaire
GWAS	Genome-wide association studies
GFR	Glomerular filtration rate
HDL	High-density lipoprotein cholesterol
HTN	Hypertension
IVW	Inverse-variance weighting
LD	Linkage disequilibrium
MR	Mendelian randomization
MR-PRESSO	Mendelian randomization pleiotropy RESidual sum and outlier
MAF	Minor allele frequency
MEC	Mobile Examination Center
NCHS	National Center for Health Statistics
NHANES	National Health and Nutrition Examination Survey
OR	Odds ratio
PIR	Poverty income ratio
RCTs	Randomized controlled trials
MR-RAPS	Robust adjusted profile score
SCR	Serum creatinine
SNPs	Single-nucleotide polymorphisms
SEM	Standard error of the mean
SE	Standard errors
SBP	Systolic blood pressure
TG	Triglycerides
UKB	UK Biobank
UACR	Urinary albumin-to-creatinine ratio
VEGF	Vascular endothelial growth factor
WME	Weighted median estimator
