# Supplementary material for: Linking artificial sweetener intake with kidney function: insights from NHANES 2003–2006 and findings from Mendelian randomization research
Source: Front Nutr. 2024 May 30;11:1387676. doi: 10.3389/fnut.2024.1387676 (PMC11169671; doi:10.3389/fnut.2024.1387676)
Supplement: Supplementary file 1 [file Data_Sheet_1.zip › Data Sheet 1/Supplement figures and tables/Supplementary figure legends.docx]

**Supplementary Figure 1.** Funnel plots of causal estimates of exposure (Intake of artificial sweetener added to cereal) on outcomes. A: chronic kidney disease; B: eGFR that uses serum cystatin C; C: GFR estimated with the use of creatinine; D: serum creatinine; E: serum cystatin C; F: urine albumin-to-creatinine ratio; G: Blood Urea Nitrogen.

**Supplementary Figure 2.** Leave-one-out stability tests causal estimates of exposure (Intake of artificial sweetener added to cereal) on outcomes. A: chronic kidney disease; B: eGFR that uses serum cystatin C; C: GFR estimated with the use of creatinine; D: serum creatinine; E: serum cystatin C; F: urine albumin-to-creatinine ratio; G: Blood Urea Nitrogen.

**Supplementary Figure 3.** Radial plots of causal estimates of exposure (Intake of artificial sweetener added to cereal) on outcomes. A: chronic kidney disease; B: eGFR that uses serum cystatin C; C: GFR estimated with the use of creatinine; D: serum creatinine; E: serum cystatin C; F: urine albumin-to-creatinine ratio; G: Blood Urea Nitrogen.

**Supplementary Figure 4.** Scatter plots of causal estimates of exposure (Intake of artificial sweetener added to cereal) on outcomes. A: chronic kidney disease; B: eGFR that uses serum cystatin C; C: GFR estimated with the use of creatinine; D: serum creatinine; E: serum cystatin C; F: urine albumin-to-creatinine ratio; G: Blood Urea Nitrogen.

**Supplementary Figure 5.** Funnel plots of causal estimates of exposure (Intake of artificial sweetener added to coffee) on outcomes. A: chronic kidney disease; B: eGFR that uses serum cystatin C; C: GFR estimated with the use of creatinine; D: serum creatinine; E: serum cystatin C; F: urine albumin-to-creatinine ratio; G: Blood Urea Nitrogen.

**Supplementary Figure 6.** Leave-one-out stability tests causal estimates of exposure (Intake of artificial sweetener added to coffee) on outcomes. A: chronic kidney disease; B: eGFR that uses serum cystatin C; C: GFR estimated with the use of creatinine; D: serum creatinine; E: serum cystatin C; F: urine albumin-to-creatinine ratio; G: Blood Urea Nitrogen.

**Supplementary Figure 7.** Radial plots of causal estimates of exposure (Intake of artificial sweetener added to coffee) on outcomes. A: chronic kidney disease; B: eGFR that uses serum cystatin C; C: GFR estimated with the use of creatinine; D: serum creatinine; E: serum cystatin C; F: urine albumin-to-creatinine ratio; G: Blood Urea Nitrogen.

**Supplementary Figure 8.** Scatter plots of causal estimates of exposure (Intake of artificial sweetener added to coffee) on outcomes. A: chronic kidney disease; B: eGFR that uses serum cystatin C; C: GFR estimated with the use of creatinine; D: serum creatinine; E: serum cystatin C; F: urine albumin-to-creatinine ratio; G: Blood Urea Nitrogen.

**Supplementary Figure 9.** Funnel plots of causal estimates of exposure (Intake of artificial sweetener added to tea) on outcomes. A: chronic kidney disease; B: eGFR that uses serum cystatin C; C: GFR estimated with the use of creatinine; D: serum creatinine; E: serum cystatin C; F: urine albumin-to-creatinine ratio; G: Blood Urea Nitrogen.

**Supplementary Figure 10.** Leave-one-out stability tests causal estimates of exposure (Intake of artificial sweetener added to tea) on outcomes. A: chronic kidney disease; B: eGFR that uses serum cystatin C; C: GFR estimated with the use of creatinine; D: serum creatinine; E: serum cystatin C; F: urine albumin-to-creatinine ratio; G: Blood Urea Nitrogen.

**Supplementary Figure 11.** Radial plots of causal estimates of exposure (Intake of artificial sweetener added to tea) on outcomes. A: chronic kidney disease; B: eGFR that uses serum cystatin C; C: GFR estimated with the use of creatinine; D: serum creatinine; E: serum cystatin C; F: urine albumin-to-creatinine ratio; G: Blood Urea Nitrogen.

**Supplementary Figure 12.** Scatter plots of causal estimates of exposure (Intake of artificial sweetener added to tea) on outcomes. A: chronic kidney disease; B: eGFR that uses serum cystatin C; C: GFR estimated with the use of creatinine; D: serum creatinine; E: serum cystatin C; F: urine albumin-to-creatinine ratio; G: Blood Urea Nitrogen.
