# Supplementary material for: Linking artificial sweetener intake with kidney function: insights from NHANES 2003–2006 and findings from Mendelian randomization research
Source: Front Nutr. 2024 May 30;11:1387676. doi: 10.3389/fnut.2024.1387676 (PMC11169671; doi:10.3389/fnut.2024.1387676)
Supplement: Supplementary file 1 [file Data_Sheet_1.zip › Data Sheet 1/Supplement figures and tables/Supplement_tables.docx]

| **Characteristics** | | **no addition**  **(n = 179)** | **occasional addition**  **(n =25)** | **regular addition**  **(n = 24)** | **frequent addition**  **(n = 12)** | **p-Value** |
| --- | --- | --- | --- | --- | --- | --- |
| Sex (n,%) | Men | 97 (54.2) | 12 (48.0) | 16 (66.7) | 10 (83.3) | 0.076 |
|  | Women | 82 (45.8) | 13 (52.0) | 8 (33.3) | 2 (16.7) |  |
| Age, years (mean, se) | | 49.03±1.74 | 46.60±4.55 | 53.00±4.18 | 65.17±5.25 | 0.040 |
| Race/ Ethnicity (n,%) | Mexican American | 30 (16.8) | 5 (20.0) | 4 (16.7) | 3 (25.0) | 0.876 |
|  | Other Hispanic | 0 (0.0) | 0 (0.0) | 1 (4.2) | 0 (0.0) |  |
|  | Non-Hispanic White | 116(64.8) | 16 (64.0) | 15 (62.5) | 8 (66.7) |  |
|  | Non-Hispanic Black | 27 (15.1) | 4 (16.0) | 3 (12.5) | 0 (0.0) |  |
|  | Other race including multiracial | 6 (3.4) | 0 (0.0) | 1 (4.2) | 1 (8.3) |  |
| Education level (n,%) | Less Than 9th Grade | 24 (13.4) | 2 (8.0) | 2 (8.3) | 6 (50.0) | 0.091 |
|  | 9-11th Grade (Includes 12th grade with no diploma) | 27 (15.1) | 3 (12.0) | 4 (16.7) | 1 (8.3) |  |
|  | High School Grad/GED or Equivalent | 39 (21.8) | 5 (20.0) | 4 (16.7) | 2 (16.7) |  |
|  | Some College or AA degree | 44 (24.6) | 6 (24.0) | 5 (20.8) | 1 (8.3) |  |
|  | College Graduate or above | 45 (25.1) | 9 (36.0) | 9 (37.5) | 2 (16.7) |  |
| Income to poverty (mean, se) | | 2.57±0.12 | 3.17±0.33 | 3.37±0.31 | 2.28±0.40 | 0.048 |
| Hypertension (n,%) | Yes | 58 (32.4) | 10 (40.0) | 11 (45.8) | 6 (50.0) | 0.442 |
|  | No | 121 (67.6) | 15 (60.0) | 13 (54.2) | 6 (50.0) |  |
| Diabetes (n,%) | Yes | 5 (2.8) | 0 (0.0) | 5 (20.8) | 3 (25.0) | <0.001 |
|  | No | 174 (97.2) | 25 (100.0） | 19 (79.2) | 9 (75.0) |  |
| Smoking (n,%) | Yes | 97 (54.2) | 11 (44.0) | 10 (41.7) | 8 (66.7) | 0.421 |
|  | No | 82 (45.8) | 14 (56.0) | 14 (58.3) | 4 (33.3) |  |
| Alcohol drinking (n,%) | Yes | 118 (65.9) | 20 (80.0) | 17 (70.8) | 7 (58.3) | 0.420 |
|  | No | 61 (34.1) | 5 (20.0) | 7 (29.2) | 5 (41.7) |  |
| Body mass index (kg/m^2^) (mean, se) | | 27.63±0.43 | 28.34±1.11 | 28.30±1.18 | 30.12±1.68 | 0.520 |
| Serum Triglycerides (mmol/L) (mean, se) | | 1.62±0.07 | 1.63±0.19 | 1.74±0.18 | 1.59±0.19 | 0.940 |
| Serum High density lipoprotein (mmol/L) (mean, se) | | 1.39±0.03 | 1.49±0.08 | 1.24±0.06 | 1.23±0.07 | 0.028 |
| Fasting blood glucose (mmol/L) (mean, se) | | 5.26±0.09 | 4.87±0.12 | 5.85±0.29 | 6.63±0.66 | 0.003 |
| Urinary albumin creatinine ratio (mg/g) (mean, se) | | 33.83±13.27 | 9.75±1.55 | 16.94±10.01 | 19.74±6.54 | 0.156 |
| Serum creatinine (umol/L) (mean, se) | | 79.81±1.72 | 73.55±4.78 | 83.24±5.05 | 88.40±5.55 | 0.245 |
| Serum Beta 2 Microglobulin (mg/L) (mean, se) | | 2.10±0.05 | 1.94±0.10 | 2.17±0.17 | 2.43±0.16 | 0.092 |
| Serum cyscatin C (mg/L) (mean, se) | | 0.78±0.01 | 0.72±0.04 | 0.82±0.05 | 0.90±0.06 | 0.071 |
| eGFRcr-cys (ml/min/1.73 m^2^) (mean, se) | | 73.55±0.64 | 76.31±1.66 | 75.95±1.78 | 72.53±2.24 | 0.287 |

Supplementary Table 1. Variable analysis of different artificial sweetener intake in the suboptimal health research population

Value expressed as mean and se or percentage. CKD, chronic kidney disease; eGFRcr-cys, based on creatinine and cystatin C estimated glomerular filtration rate.

Supplementary Table 2. Association of genetically determined Artificial Sweeteners with eGFRcysc

| **Outcome** | **Exposure** | **No.of SNPs used** | **F-statistic^*^** | **Mendelian randomization method** | **OR(95% CI)** | **P-value** | **adjusted P- value^*^** | **Cochran’s Q (I2)** | **Q-value** | **MR-Egger intercept (P value)** | **outliers from**  **MR-presso** | **Outliers from**  **MR-radial** |
| --- | --- | --- | --- | --- | --- | --- | --- | --- | --- | --- | --- | --- |
| eGFRcysc | artificial sweetener added to cereal | 26 | 91.88 | Inverse-variance weighted | 1.02(1.00-1.04) | 0.017 | 0.057 | 21.0(0.00%) | 0.691 | -0.0001(0.687) | NA | NA |
|  |  |  |  | MR Egger method | 1.03(0.99-1.08) | 0.152 | 0.213 | 20.9(0.00%) | 0.647 |  |  |  |
|  |  |  |  | Weighted median | 1.03(1.00-1.06) | 0.031 | 0.062 |  |  |  |  |  |
|  |  |  |  | MR-RAPS | 1.03(1.00-1.05) | 0.019 | 0.057 |  |  |  |  |  |
|  | artificial sweetener added to tea | 21 | 211.36 | Inverse-variance weighted | 1.00(0.99-1.01) | 0.779 | 0.783 | 14.4(0.00%) | 0.812 | -0.0008(0.088) | NA | NA |
|  |  |  |  | MR Egger method | 1.03(0.99-1.06) | 0.135 | 0.638 | 11.1(0.00%) | 0.920 |  |  |  |
|  |  |  |  | Weighted median | 1.01(0.99-1.03) | 0.382 | 0.638 |  |  |  |  |  |
|  |  |  |  | MR-RAPS | 1.00(0.99-1.01) | 0.783 | 0.783 |  |  |  |  |  |
|  | artificial sweetener added to coffee | 20 | 229.34 | Inverse-variance weighted | 1.00(0.99-1.01) | 0.983 | 0.983 | 15.0(0.00%) | 0.725 | 0.0003(0.482) | NA | NA |
|  |  |  |  | MR Egger method | 0.99(0.97-1.02) | 0.537 | 0.805 | 14.4(0.00%) | 0.699 |  |  |  |
|  |  |  |  | Weighted median | 0.99(0.98-1.01) | 0.405 | 0.804 |  |  |  |  |  |
|  |  |  |  | MR-RAPS | 1.00(0.99-1.01) | 0.983 | 0.983 |  |  |  |  |  |

MR-PRESSO, Mendelian randomization pleiotropy residual sum and outlier; CKD, chronic kidney disease; OR, odds ratio; CI, confidence interval; MR-RAPS, Robust adjusted profile score; adjusted P- value, The P-values are adjusted according to the Benjamin-Hochberg method; The mean F statistics of the instruments were calculated for IVW, WM and MR-Egger.

Supplementary Table 3. Association of genetically determined Artificial Sweeteners with eGFRcr

| **outco**me | **exposure** | **No.of SNPs used** | **F-statistic^*^** | **mendelian randomization method** | **OR(95% CI)** | **P-value** | **adjusted P- value^*^** | **cochran’sQ (I2)** | **Q-value** | **MR-Egger intercept (P value)** | **outliers from**  **MR-presso** | **Outliers from**  **MR-radial** | | |
| --- | --- | --- | --- | --- | --- | --- | --- | --- | --- | --- | --- | --- | --- | --- |
| eGFRcr | artificial sweetener added to cereal | 23 | 91.24 | Inverse-variance weighted | 1.01(1.00-1.02) | 0.073 | 0.248 | 14.5(0.00%) | 0.881 | 0.0001(0.494) | rs117206618  rs2288674  rs61117377  rs9807368 | rs143055801  rs75209402 | | |
|  |  |  |  | MR Egger method | 1.00(0.98-1.03) | 0.809 | 0.809 | 14.1(0.00%) | 0.867 |  |  | |  |  |
|  |  |  |  | Weighted median | 1.01(0.99-1.03) | 0.185 | 0.271 |  |  |  |  | |  |  |
|  |  |  |  | MR-RAPS | 1.01(1.00-1.03) | 0.083 | 0.248 |  |  |  |  | |  |  |
|  | artificial sweetener added to tea | 26 | 214.97 | Inverse-variance weighted | 1.00(0.99-1.01) | 0.504 | 0.605 | 27.8(10.18%) | 0.316 | -0.00004(0.854) | NA | | rs57624958 |  |
|  |  |  |  | MR Egger method | 1.00(0.98-1.02) | 0.905 | 0.905 | 27.8(10.18%) | 0.269 |  |  | |  |  |
|  |  |  |  | Weighted median | 1.00(0.99-1.01) | 0.437 | 0.605 |  |  |  |  | |  |  |
|  |  |  |  | MR-RAPS | 1.00(0.99-1.01) | 0.478 | 0.605 |  |  |  |  | |  |  |
|  | artificial sweetener added to coffee | 18 | 230.69 | Inverse-variance weighted | 1.00(0.99-1.01) | 0.631 | 0.767 | 10.4(0.00%) | 0.847 | -0.0001(0.664) | rs11068069  rs1181772  rs11936086  rs59576512  rs12270786 | | rs72907976 |  |
|  |  |  |  | MR Egger method | 1.00(0.99-1.02) | 0.887 | 0.887 | 10.2(0.00%) | 0.809 |  |  | |  |  |
|  |  |  |  | Weighted median | 1.00(0.99-1.01) | 0.481 | 0.767 |  |  |  |  | |  |  |
|  |  |  |  | MR-RAPS | 1.00(0.99-1.01) | 0.640 | 0.767 |  |  |  |  | |  |  |

MR-PRESSO, Mendelian randomization pleiotropy residual sum and outlier; CKD, chronic kidney disease; OR, odds ratio; CI, confidence interval; MR-RAPS, Robust adjusted profile score; adjusted P- value, The P-values are adjusted according to the Benjamin-Hochberg method; The mean F statistics of the instruments were calculated for IVW, WM and MR-Egger.

Supplementary Table 4. Association of genetically determined Artificial Sweeteners with SCR

| Outcome | Exposure | | No.of SNPs used | | F-statistic^*^ | | Mendelian Randomization Method | | OR(95% CI) | | P-value | | | adjusted P- value^*^ | | | | | Cochran’sQ (I2) | | | | Q-value | | | | MR-Egger intercept (P value) | | | | Outliers from  MR-PRESSO | | | | Outliers from  MR-Radial | | |
| --- | --- | --- | --- | --- | --- | --- | --- | --- | --- | --- | --- | --- | --- | --- | --- | --- | --- | --- | --- | --- | --- | --- | --- | --- | --- | --- | --- | --- | --- | --- | --- | --- | --- | --- | --- | --- | --- |
| SCR | artificial sweetener added to cereal | | 27 | | 91.57 | | Inverse-variance weighted | | 0.91(0.85-0.98) | | 0.017 | | | 0.056 | | | 23.1(0.00%) | | | | 0.629 | | | | -0.001(0.332) | | | | rs75209402  rs61117377 | | | | rs118020038 | | | |  |
|  |  | |  | |  | | MR Egger method | | 0.98(0.84-1.14) | | 0.775 | | 0.775 | | | 22.1(0.00%) | | | | 0.631 | | | |  | | | |  | | | |  | | | |  |  |
|  | |  | |  | |  | | Weighted median | | 0.91(0.82-1.01) | | 0.087 | | | 0.128 | | |  | | | |  | | | |  | | | |  | | | |  | | |  |
|  | |  | |  | |  | | MR-RAPS | | 0.91(0.84-0.98) | | 0.019 | | | 0.056 | | |  | | | |  | | | |  | | | |  | | | |  | | |  |
|  | | artificial sweetener added to tea | | 24 | | 216.45 | | Inverse-variance weighted | | 0.99(0.95-1.04) | | 0.751 | | | 0.756 | | | 21.1(0.00%) | | | | 0.573 | | | | -0.0008(0.621) | | | | rs74372881  rs35172567 | | | | rs10505568  rs1335579 | | |  |
|  | |  | |  | |  | | MR Egger method | | 1.02(0.91-1.13) | | 0.756 | | | 0.756 | | | 20.9(0.00%) | | | | 0.528 | | | |  | | | |  | | | |  | | |  |
|  | |  | |  | |  | | Weighted median | | 0.96(0.89-1.03) | | 0.216 | | | 0.623 | | |  | | | |  | | | |  | | | |  | | | |  | | |  |
|  | |  | |  | |  | | MR-RAPS | | 0.99(0.95-1.04) | | 0.752 | | | 0.756 | | |  | | | |  | | | |  | | | |  | | | |  | | |  |
|  | | artificial sweetener added to coffee | | 21 | | 232.74 | | Inverse-variance weighted | | 0.98(0.93-1.02) | | 0.292 | | | 0.584 | | | 21.1(5.35%) | | | | 0.390 | | | | 0.001(0.444) | | | | rs12270786  rs72710041 | | | | rs11068069  rs59576512 | | |  |
|  | |  | |  | |  | | MR Egger method | | 0.94(0.85-1.04) | | 0.250 | | | 0.584 | | | 20.5(5.35%) | | | | 0.367 | | | |  | | | |  | | | |  | | |  |
|  | |  | |  | |  | | Weighted median | | 0.99(0.92-1.06) | | 0.726 | | | 0.868 | | |  | | | |  | | | |  | | | |  | | | |  | | |  |
|  | |  | |  | |  | | MR-RAPS | | 0.97(0.93-1.02) | | 0.279 | | | 0.584 | | |  | | | |  | | | |  | | | |  | | | |  | | |  |

MR-PRESSO, Mendelian randomization pleiotropy residual sum and outlier; CKD, chronic kidney disease; OR, odds ratio; CI, confidence interval; MR-RAPS, Robust adjusted profile score; adjusted P- value, The P-values are adjusted according to the Benjamin-Hochberg method; The mean F statistics of the instruments were calculated for IVW, WM and MR-Egger.

Supplementary Table 5. Association of genetically determined Artificial Sweeteners with CYSC

| **Outcome** | **Exposure** | **No.of SNPs used** | **F-statistic^*^** | **Mendelian Randomization Method** | **OR(95% CI)** | **P-value** | | **adjusted P- value^*^** | | **Cochran’sQ (I2)** | | **Q-value** | | **MR-Egger intercept (P value)** | | **Outliers from**  **MR-PRESSO** | | **Outliers from**  **MR-Radial** | |
| --- | --- | --- | --- | --- | --- | --- | --- | --- | --- | --- | --- | --- | --- | --- | --- | --- | --- | --- | --- |
| CYSC | artificial sweetener added to cereal | 11 | 91.12 | Inverse-variance weighted | 0.95(0.83-1.07) | 0.387 | 0.801 | | 7.1(0.00%) | | 0.720 | | 0.001(0.461) | | NA | | rs214818 | |  |
|  |  |  |  | MR Egger method | 0.86(0.65-1.14) | 0.310 | 0.801 | | 6.5(0.00%) | | 0.693 | |  | |  | |  | |  |
|  |  |  |  | Weighted median | 0.97(0.83-1.14) | 0.695 | 0.878 | |  | |  | |  | |  | |  | |  |
|  |  |  |  | MR-RAPS | 0.94(0.83-1.08) | 0.401 | 0.801 | |  | |  | |  | |  | |  | |  |
|  | artificial sweetener added to tea | 11 | 210.82 | Inverse-variance weighted | 0.99(0.92-1.06) | 0.721 | 0.870 | | 8.0(0.00%) | | 0.628 | | -0.0003(0.870) | | NA | | rs1335579 | |  |
|  |  |  |  | MR Egger method | 1.00(0.85-1.17) | 0.998 | 0.998 | | 8.0(0.00%) | | 0.536 | |  | |  | |  | |  |
|  |  |  |  | Weighted median | 0.96(0.87-1.05) | 0.358 | 0.870 | |  | |  | |  | |  | |  | |  |
|  |  |  |  | MR-RAPS | 0.99(0.92-1.06) | 0.725 | 0.870 | |  | |  | |  | |  | |  | |  |
|  | artificial sweetener added to coffee | 7 | 225.87 | Inverse-variance weighted | 0.98(0.90-1.06) | 0.612 | 0.673 | | 6.4(5.99%) | | 0.382 | | -0.003(0.390) | | NA | | NA | |  |
|  |  |  |  | MR Egger method | 1.10(0.85-1.43) | 0.493 | 0.673 | | 5.4(5.99%) | | 0.367 | |  | |  | |  | |  |
|  |  |  |  | Weighted median | 0.93(0.84-1.04) | 0.189 | 0.673 | |  | |  | |  | |  | |  | |  |
|  |  |  |  | MR-RAPS | NA | NA | NA | |  | |  | |  | |  | |  | |  |

MR-PRESSO, Mendelian randomization pleiotropy residual sum and outlier; CKD, chronic kidney disease; OR, odds ratio; CI, confidence interval; MR-RAPS, Robust adjusted profile score; adjusted P- value, The P-values are adjusted according to the Benjamin-Hochberg method; The mean F statistics of the instruments were calculated for IVW, WM and MR-Egger.

Supplementary Table 6. Association of genetically determined Artificial Sweeteners with UACR

| **Outcome** | **Exposure** | **No.of SNPs used** | **F-statistic^*^** | **Mendelian Randomization Method** | **OR(95% CI)** | **P-value** | | **adjusted P- value^*^** | | **Cochran’sQ (I2)** | | **Q-value** | | **MR-Egger intercept (P value)** | | **Outliers from**  **MR-PRESSO** | | **Outliers from**  **MR-Radial** | |
| --- | --- | --- | --- | --- | --- | --- | --- | --- | --- | --- | --- | --- | --- | --- | --- | --- | --- | --- | --- |
| UACR | artificial sweetener added to cereal | 31 | 91.32 | Inverse-variance weighted | 1.02(0.95-1.09) | 0.599 | 0.943 | | 20.7(0.00%) | | 0.896 | | 0.0006(0.557) | | NA | | rs118147801 | |  |
|  |  |  |  | MR Egger method | 0.98(0.86-1.12) | 0.803 | 0.943 | | 20.4(0.00%) | | 0.880 | |  | |  | |  | |  |
|  |  |  |  | Weighted median | 1.02(0.93-1.12) | 0.684 | 0.943 | |  | |  | |  | |  | |  | |  |
|  |  |  |  | MR-RAPS | 1.02(0.95-1.09) | 0.608 | 0.943 | |  | |  | |  | |  | |  | |  |
|  | artificial sweetener added to tea | 27 | 220.63 | Inverse-variance weighted | 1.00(0.96-1.04) | 0.976 | 0.977 | | 15.8(0.00%) | | 0.940 | | 0.0007(0.593) | | NA | | rs77014507 | |  |
|  |  |  |  | MR Egger method | 0.98(0.89-1.07) | 0.642 | 0.977 | | 15.5(0.00%) | | 0.928 | |  | |  | |  | |  |
|  |  |  |  | Weighted median | 0.99(0.93-1.05) | 0.754 | 0.977 | |  | |  | |  | |  | |  | |  |
|  |  |  |  | MR-RAPS | 1.00(0.96-1.04) | 0.977 | 0.977 | |  | |  | |  | |  | |  | |  |
|  | artificial sweetener added to coffee | 24 | 228.67 | Inverse-variance weighted | 0.94(0.90-0.98) | 0.002 | 0.009 | | 18.0(0.00%) | | 0.757 | | -0.001(0.438) | | NA | | rs141134342 | |  |
|  |  |  |  | MR Egger method | 0.97(0.89-1.05) | 0.429 | 0.429 | | 17.4(0.00%) | | 0.741 | |  | |  | |  | |  |
|  |  |  |  | Weighted median | 0.94(0.89-1.00) | 0.055 | 0.111 | |  | |  | |  | |  | |  | |  |
|  |  |  |  | MR-RAPS | 0.94(0.90-0.98) | 0.003 | 0.009 | |  | |  | |  | |  | |  | |  |

MR-PRESSO, Mendelian randomization pleiotropy residual sum and outlier; CKD, chronic kidney disease; OR, odds ratio; CI, confidence interval; MR-RAPS, Robust adjusted profile score; adjusted P- value, The P-values are adjusted according to the Benjamin-Hochberg method; The mean F statistics of the instruments were calculated for IVW, WM and MR-Egger.

Supplementary Table 7. Association of genetically determined Artificial Sweeteners with BUN

| **Outcome** | **Exposure** | **No.of SNPs used** | **F-statistic^*^** | **Mendelian Randomization Method** | **OR(95% CI)** | **P-value** | | **adjusted P- value^*^** | | **Cochran’sQ (I2)** | | **Q-value** | | **MR-Egger intercept (P value)** | | **Outliers from**  **MR-PRESSO** | | **Outliers from**  **MR-Radial** | |
| --- | --- | --- | --- | --- | --- | --- | --- | --- | --- | --- | --- | --- | --- | --- | --- | --- | --- | --- | --- |
| BUN | artificial sweetener added to cereal | 26 | 91.62 | Inverse-variance weighted | 0.91(0.84-1.00) | 0.049 | 0.152 | | 24.7(0.00%) | | 0.481 | | 0.0003(0.829) | | rs115790761  rs149157321  rs61117377 | | rs145641302 | |  |
|  |  |  |  | MR Egger method | 0.90(0.74-1.09) | 0.280 | 0.280 | | 24.6(0.00%) | | 0.426 | |  | |  | |  | |  |
|  |  |  |  | Weighted median | 0.91(0.80-1.02) | 0.116 | 0.232 | |  | |  | |  | |  | |  | |  |
|  |  |  |  | MR-RAPS | 0.91(0.83-1.00) | 0.051 | 0.152 | |  | |  | |  | |  | |  | |  |
|  | artificial sweetener added to tea | 26 | 221.33 | Inverse-variance weighted | 1.00(0.95-1.05) | 0.944 | 0.943 | | 25.6(2.51%) | | 0.427 | | -0.001(0.394) | | NA | | rs185504917  rs62045220 | |  |
|  |  |  |  | MR Egger method | 1.05(0.93-1.19) | 0.455 | 0.683 | | 24.9(2.51%) | | 0.413 | |  | |  | |  | |  |
|  |  |  |  | Weighted median | 0.95(0.88-1.03) | 0.232 | 0.518 | |  | |  | |  | |  | |  | |  |
|  |  |  |  | MR-RAPS | 1.00(0.95-1.05) | 0.943 | 0.943 | |  | |  | |  | |  | |  | |  |
|  | artificial sweetener added to coffee | 25 | 228.45 | Inverse-variance weighted | 1.01(0.96-1.06) | 0.738 | 0.894 | | 14.6(0.00%) | | 0.933 | | 0.0004(0.774) | | NA | | NA | |  |
|  |  |  |  | MR Egger method | 0.99(0.90-1.11) | 0.923 | 0.923 | | 14.5(0.00%) | | 0.913 | |  | |  | |  | |  |
|  |  |  |  | Weighted median | 0.98(0.91-1.05) | 0.520 | 0.894 | |  | |  | |  | |  | |  | |  |
|  |  |  |  | MR-RAPS | 1.01(0.96-1.06) | 0.745 | 0.894 | |  | |  | |  | |  | |  | |  |

MR-PRESSO, Mendelian randomization pleiotropy residual sum and outlier; CKD, chronic kidney disease; OR, odds ratio; CI, confidence interval; MR-RAPS, Robust adjusted profile score; adjusted P- value, The P-values are adjusted according to the Benjamin-Hochberg method; The mean F statistics of the instruments were calculated for IVW, WM and MR-Egger.
