# Supplementary figures and images for: Linking artificial sweetener intake with kidney function: insights from NHANES 2003–2006 and findings from Mendelian randomization research
Source: Front Nutr. 2024 May 30;11:1387676. doi: 10.3389/fnut.2024.1387676 (PMC11169671; doi:10.3389/fnut.2024.1387676)

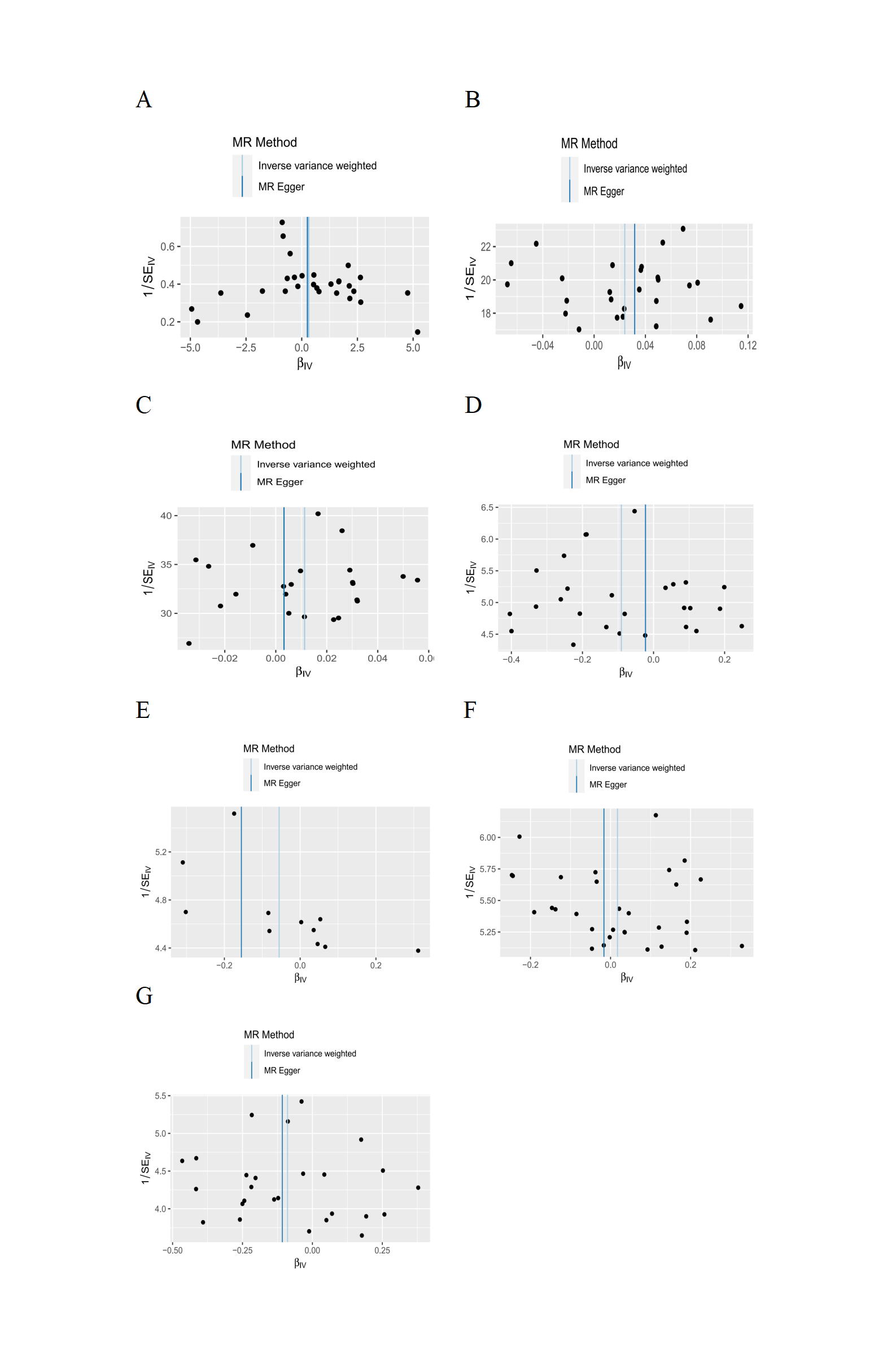

Supplement: Supplementary file 1 [file Data_Sheet_1.zip › Data Sheet 1/Supplement figures and tables/Supplementary Figure 1.png]

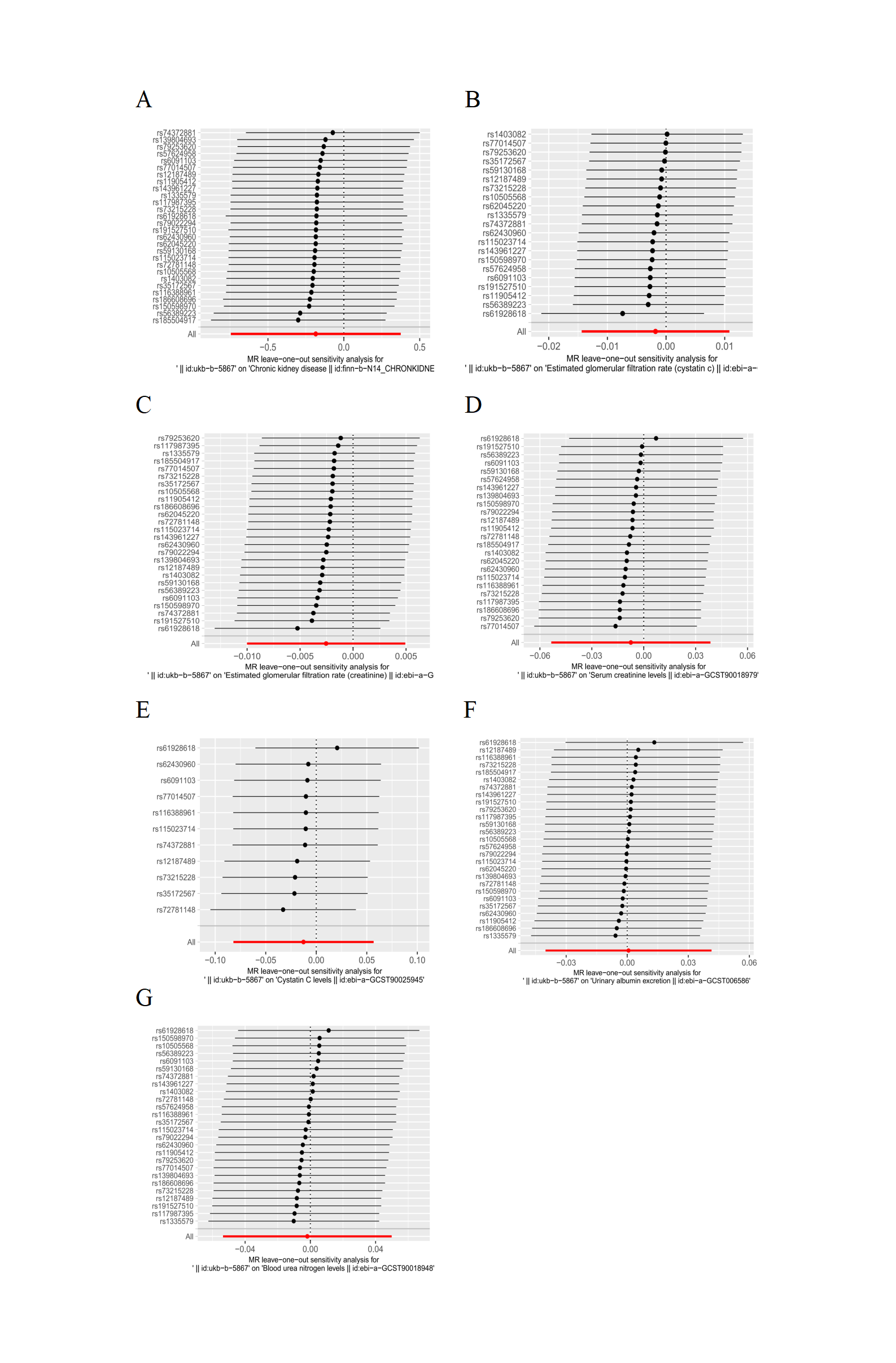

Supplement: Supplementary file 1 [file Data_Sheet_1.zip › Data Sheet 1/Supplement figures and tables/Supplementary Figure 10.png]

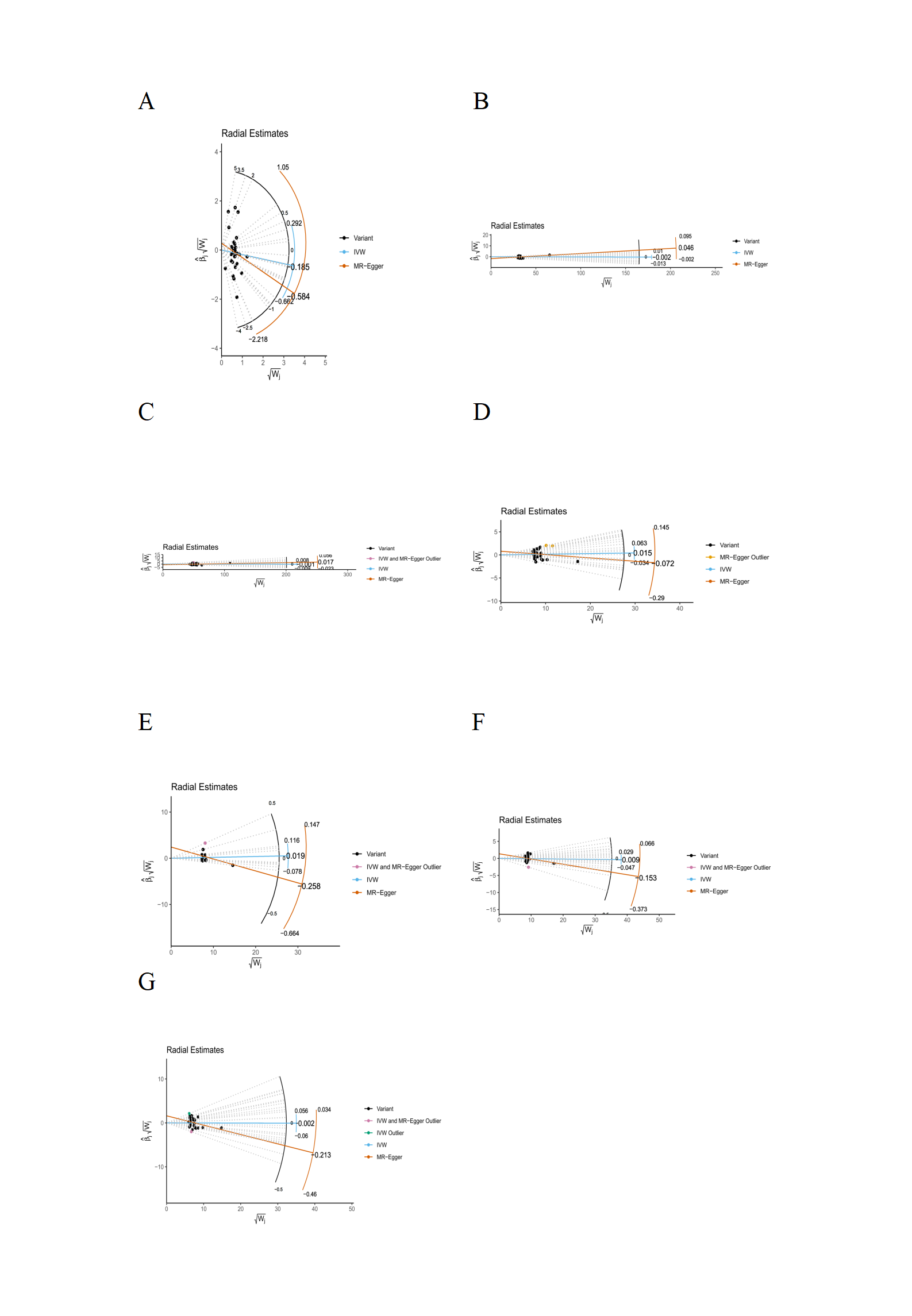

Supplement: Supplementary file 1 [file Data_Sheet_1.zip › Data Sheet 1/Supplement figures and tables/Supplementary Figure 11.png]

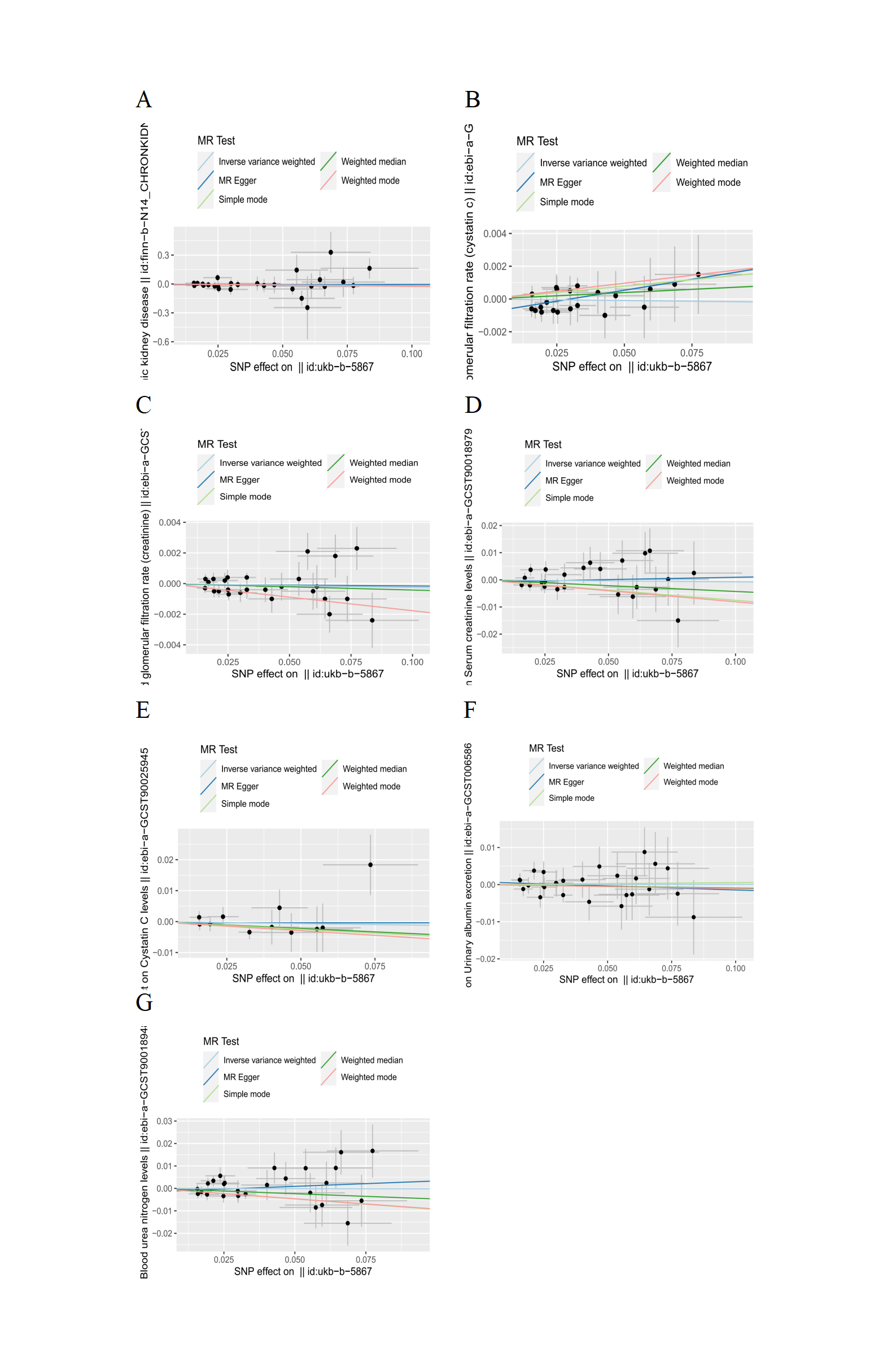

Supplement: Supplementary file 1 [file Data_Sheet_1.zip › Data Sheet 1/Supplement figures and tables/Supplementary Figure 12.png]

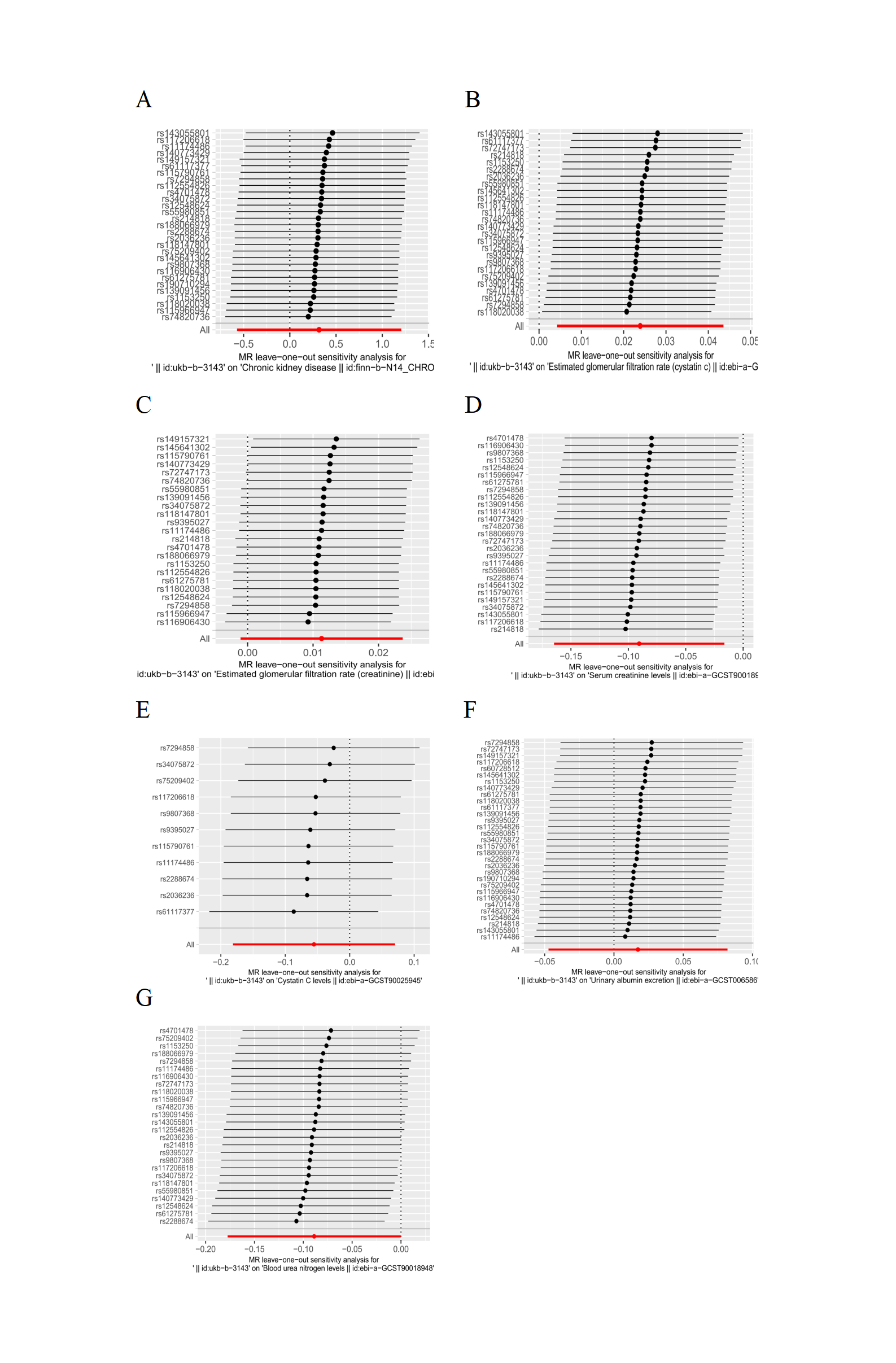

Supplement: Supplementary file 1 [file Data_Sheet_1.zip › Data Sheet 1/Supplement figures and tables/Supplementary Figure 2.png]

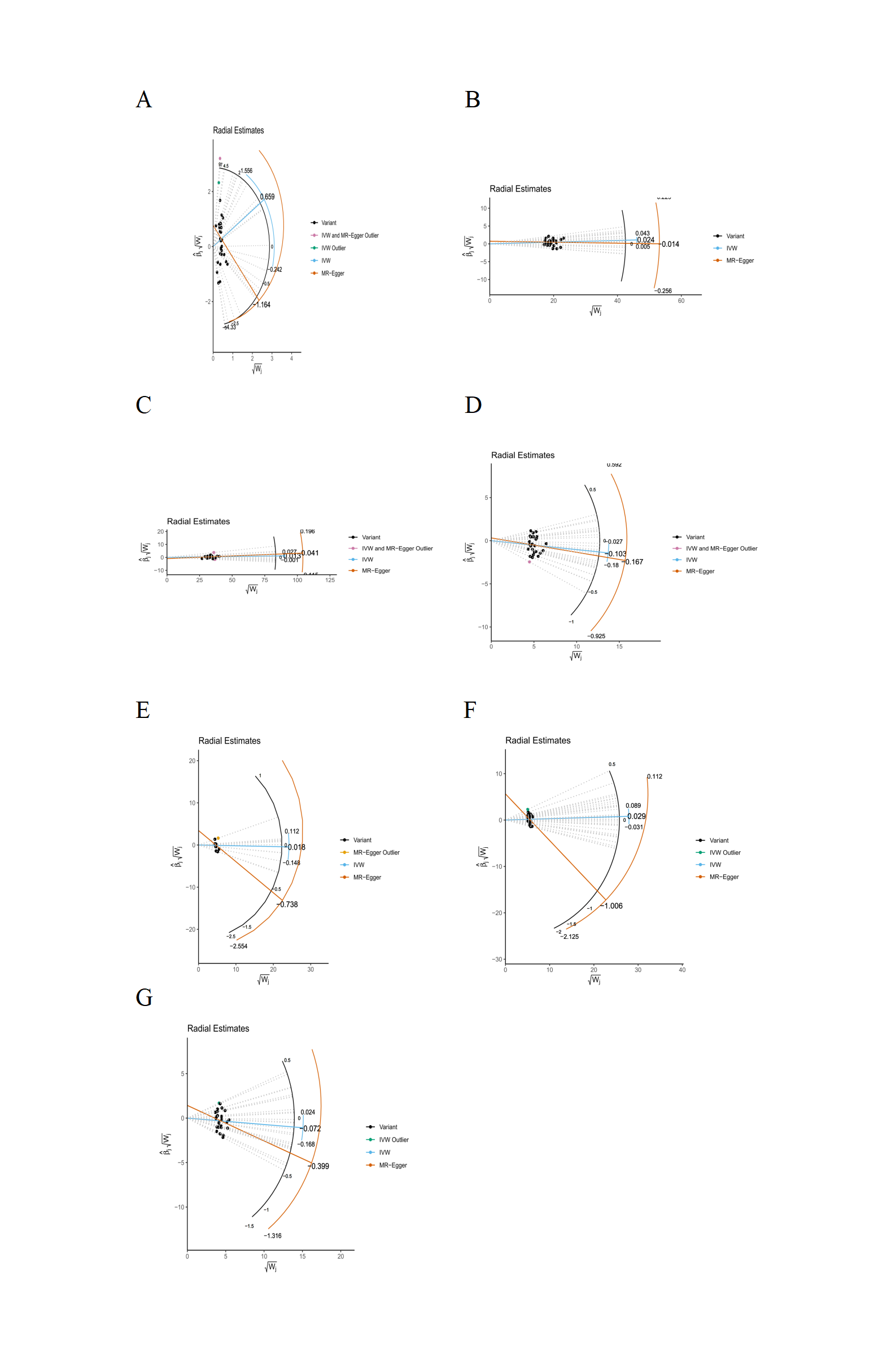

Supplement: Supplementary file 1 [file Data_Sheet_1.zip › Data Sheet 1/Supplement figures and tables/Supplementary Figure 3.png]

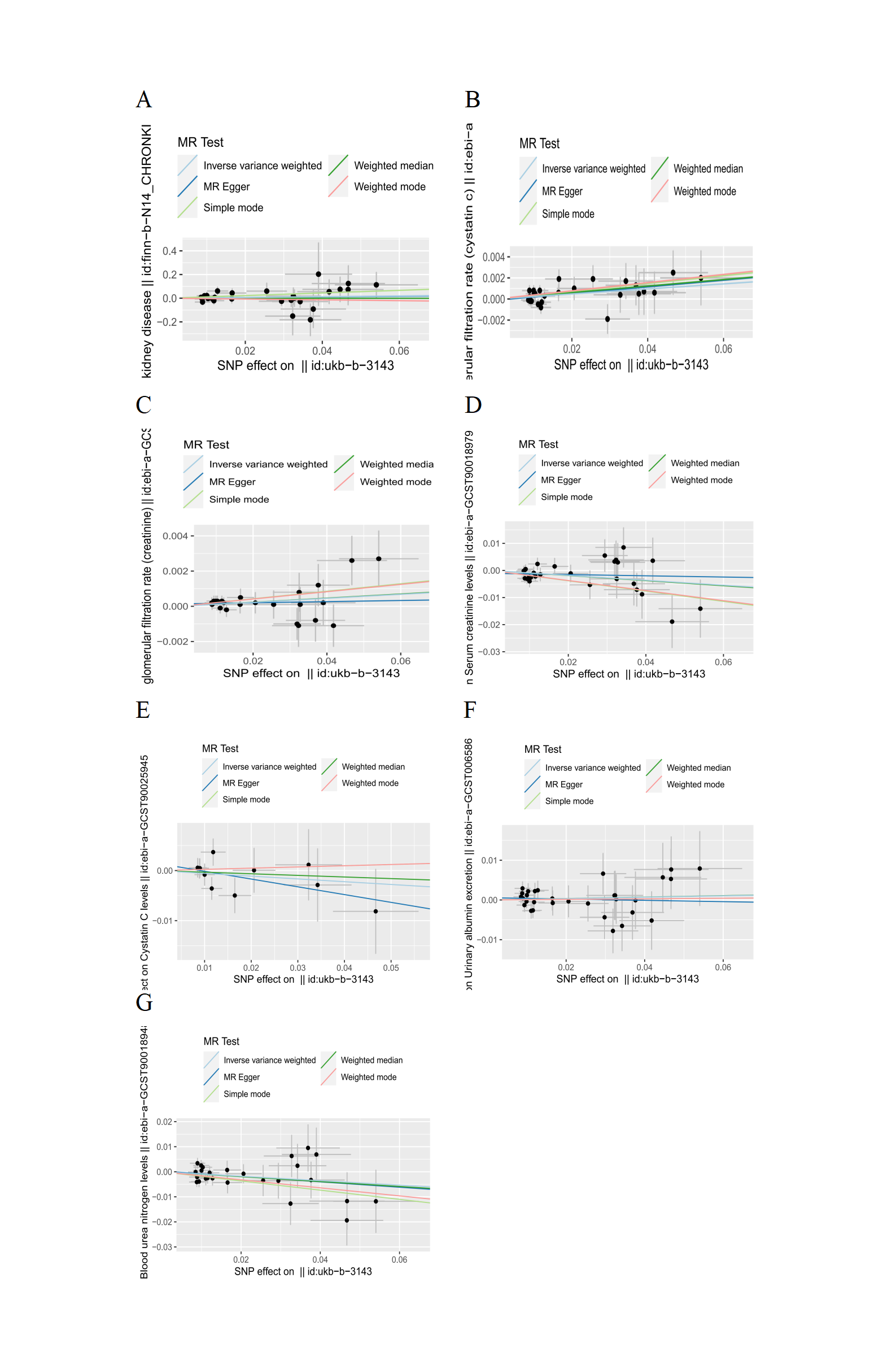

Supplement: Supplementary file 1 [file Data_Sheet_1.zip › Data Sheet 1/Supplement figures and tables/Supplementary Figure 4.png]

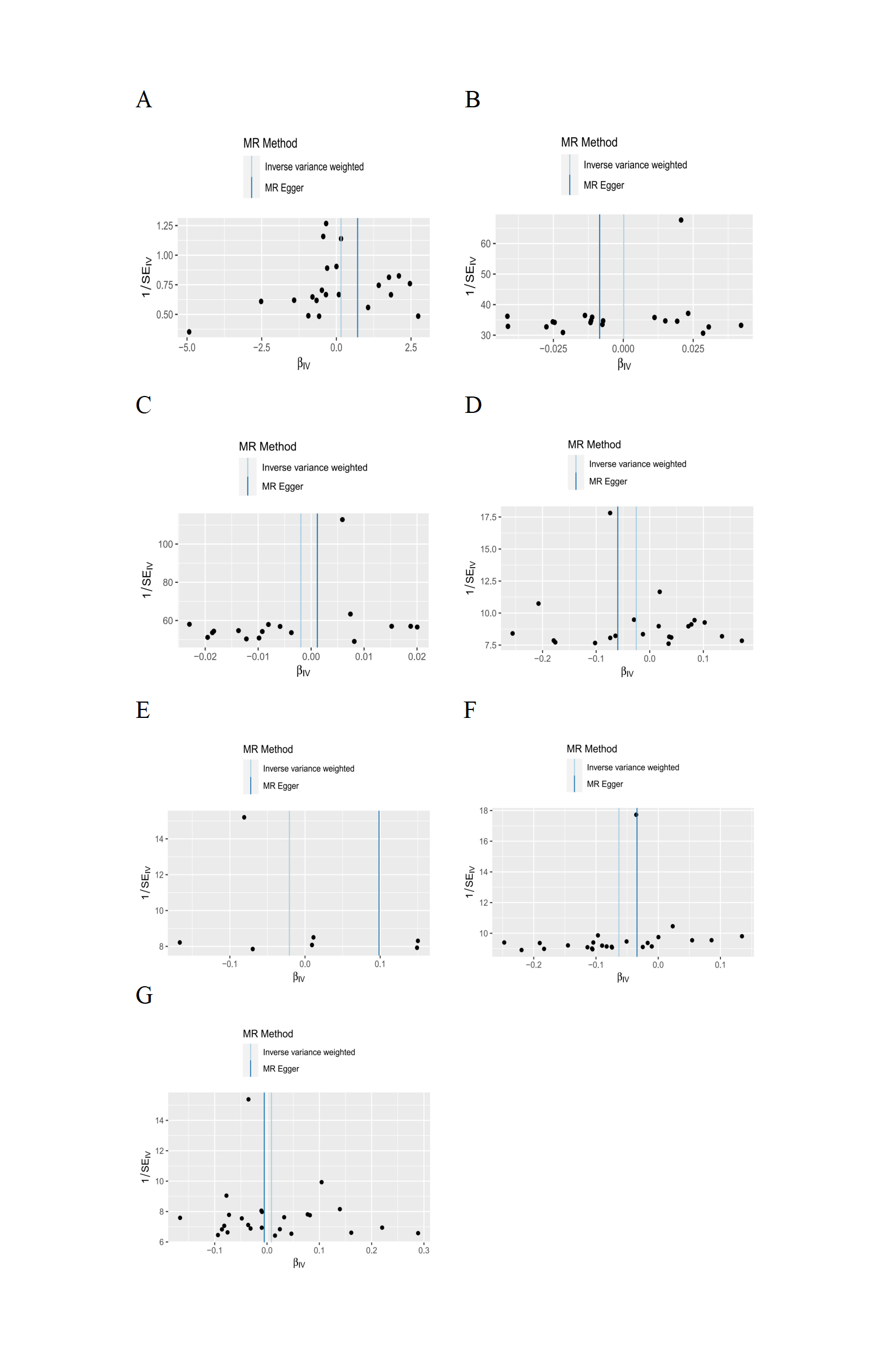

Supplement: Supplementary file 1 [file Data_Sheet_1.zip › Data Sheet 1/Supplement figures and tables/Supplementary Figure 5.png]

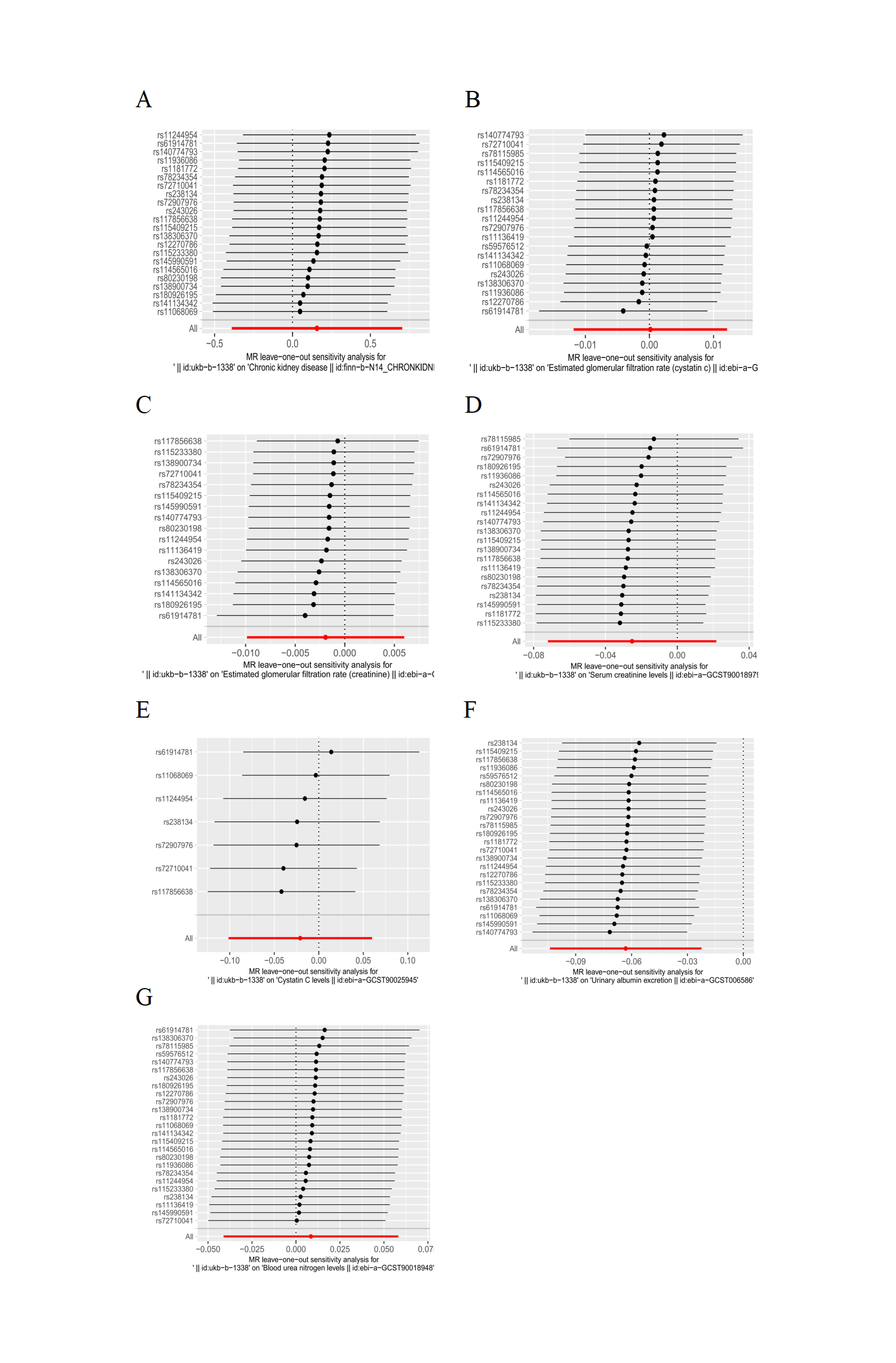

Supplement: Supplementary file 1 [file Data_Sheet_1.zip › Data Sheet 1/Supplement figures and tables/Supplementary Figure 6.png]

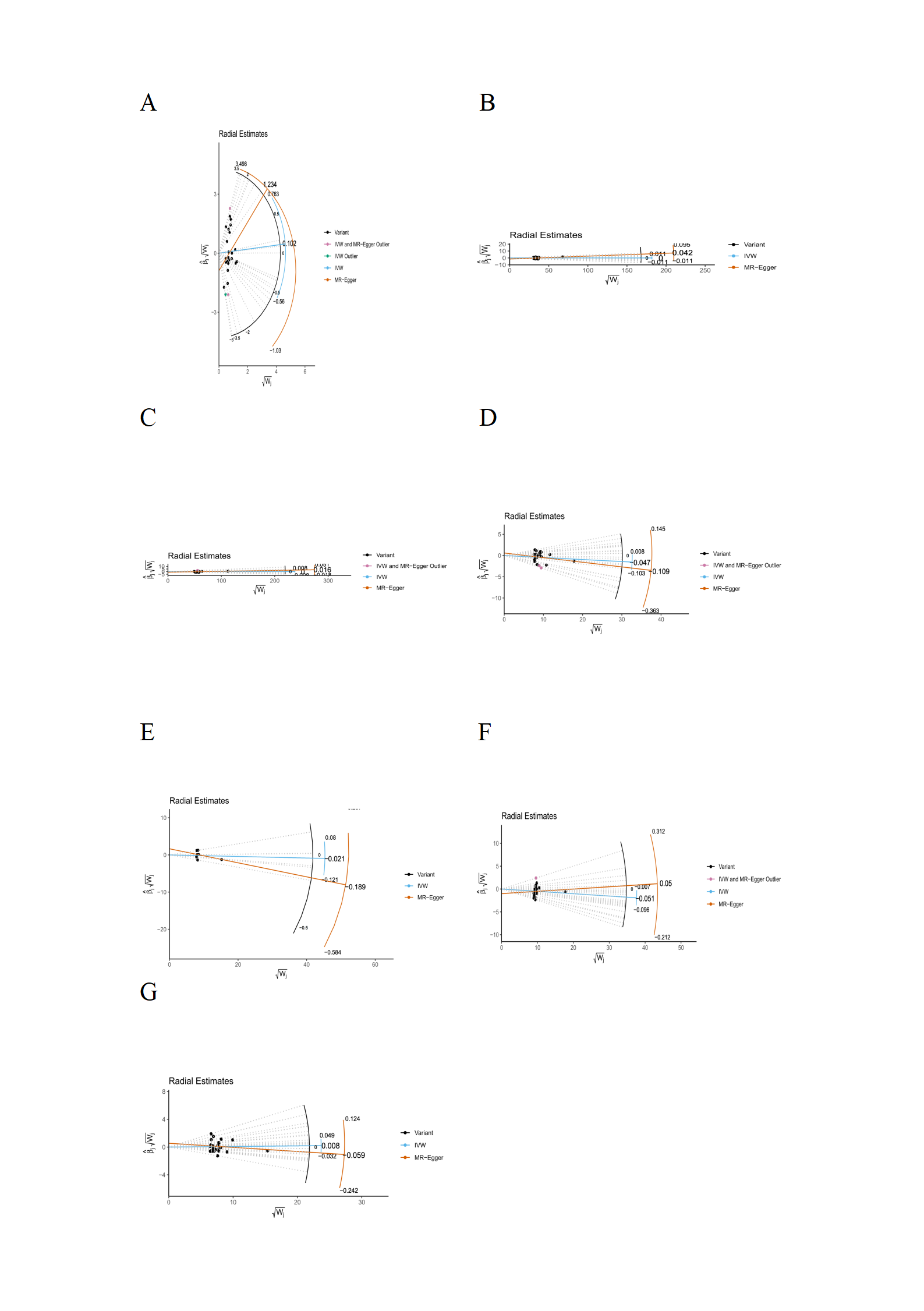

Supplement: Supplementary file 1 [file Data_Sheet_1.zip › Data Sheet 1/Supplement figures and tables/Supplementary Figure 7.png]

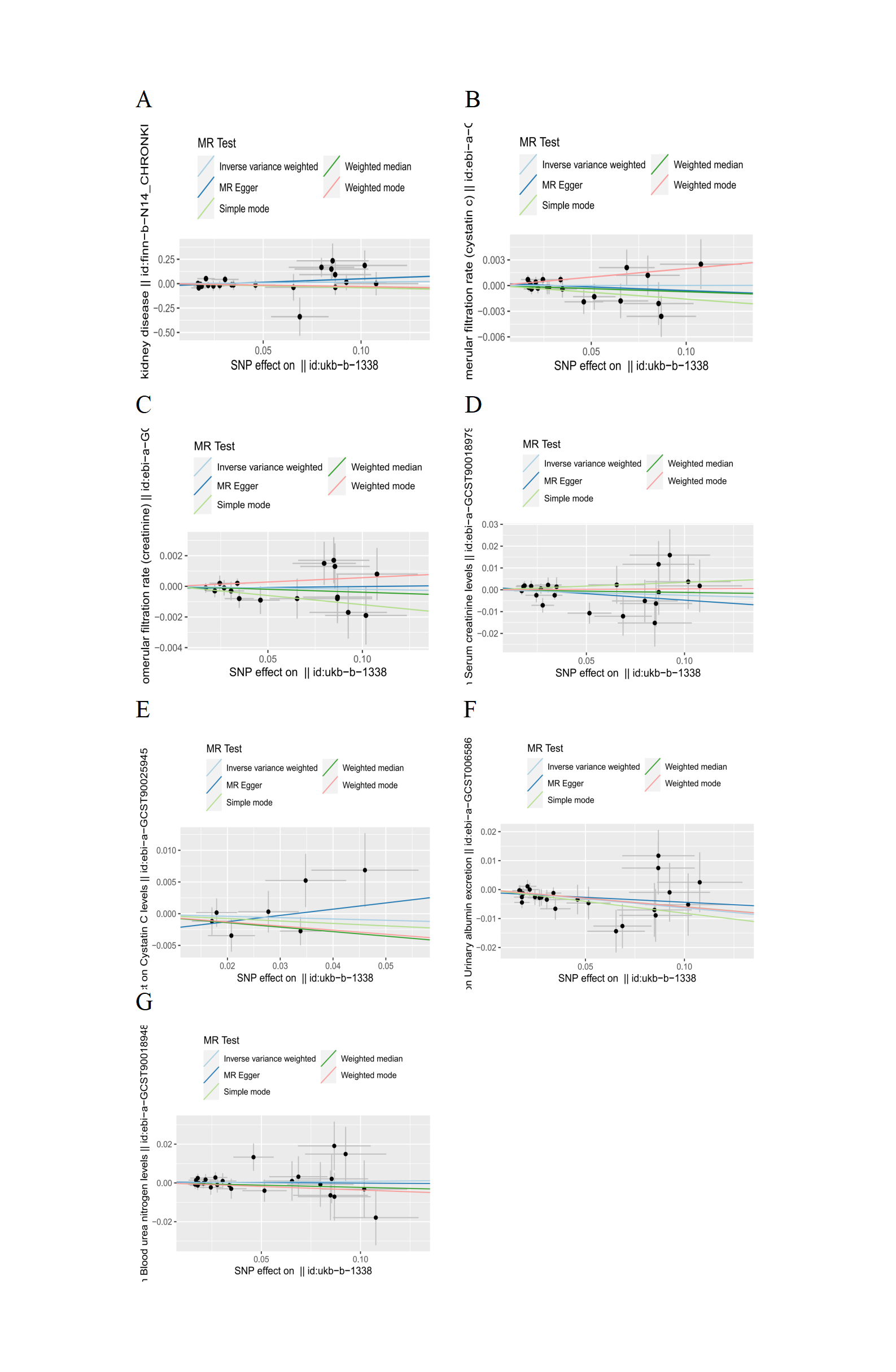

Supplement: Supplementary file 1 [file Data_Sheet_1.zip › Data Sheet 1/Supplement figures and tables/Supplementary Figure 8.png]

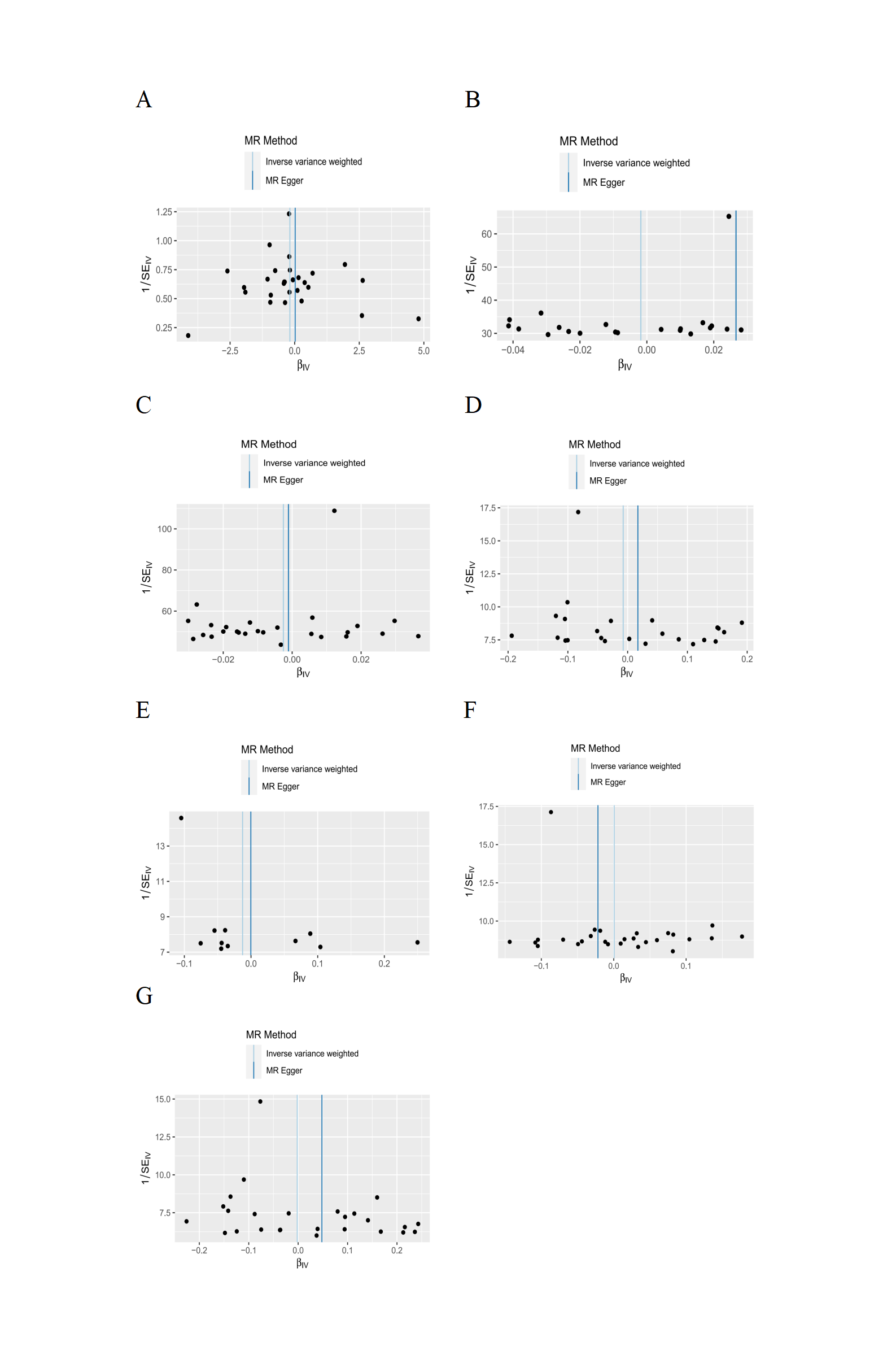

Supplement: Supplementary file 1 [file Data_Sheet_1.zip › Data Sheet 1/Supplement figures and tables/Supplementary Figure 9.png]
